# iRUNNER: A Baseline Mutation Burden Regression for Identifying Gene Interaction Between Rare Variants for Diseases

**DOI:** 10.1093/gpbjnl/qzaf135

**Published:** 2025-12-30

**Authors:** Hui Jiang, Bin Tang, Kun Li, Liubin Zhang, Junhao Liang, Clara Sze-Man Tang, Paul Kwong-Hang Tam, Binbin Wang, Youqiang Song, Qiang Wang, Mulin Jun Li, Hailiang Huang, Miaoxin Li

**Affiliations:** Zhongshan School of Medicine, Sun Yat-sen University, Guangzhou 510080, China; Department of Medical Genetics and Prenatal Diagnosis, The Third Affiliated Hospital of Zhengzhou University, Zhengzhou 450052, China; Key Laboratory of Tropical Disease Control (Sun Yat-sen University), Ministry of Education, Sun Yat-sen University, Guangzhou 510080, China; Center for Precision Medicine, Sun Yat-sen University, Guangzhou 510080, China; Zhongshan School of Medicine, Sun Yat-sen University, Guangzhou 510080, China; Key Laboratory of Tropical Disease Control (Sun Yat-sen University), Ministry of Education, Sun Yat-sen University, Guangzhou 510080, China; Center for Precision Medicine, Sun Yat-sen University, Guangzhou 510080, China; Zhongshan School of Medicine, Sun Yat-sen University, Guangzhou 510080, China; Zhongshan School of Medicine, Sun Yat-sen University, Guangzhou 510080, China; Key Laboratory of Tropical Disease Control (Sun Yat-sen University), Ministry of Education, Sun Yat-sen University, Guangzhou 510080, China; Center for Precision Medicine, Sun Yat-sen University, Guangzhou 510080, China; Zhongshan School of Medicine, Sun Yat-sen University, Guangzhou 510080, China; Key Laboratory of Tropical Disease Control (Sun Yat-sen University), Ministry of Education, Sun Yat-sen University, Guangzhou 510080, China; Center for Precision Medicine, Sun Yat-sen University, Guangzhou 510080, China; Department of Surgery, The University of Hong Kong, Hong Kong Special Administrative Region 999077, China; Dr. Li Dak-Sum Research Centre, The University of Hong Kong–Karolinska Institute Collaboration in Regenerative Medicine, Hong Kong Special Administrative Region 999077, China; Department of Surgery, The University of Hong Kong, Hong Kong Special Administrative Region 999077, China; Dr. Li Dak-Sum Research Centre, The University of Hong Kong–Karolinska Institute Collaboration in Regenerative Medicine, Hong Kong Special Administrative Region 999077, China; Faculty of Medicine, Macau University of Science and Technology, Macau Special Administrative Region 999078, China; Department of Genetics, National Research Institute for Family Planning, Beijing 100081, China; School of Biomedical Sciences, The University of Hong Kong, Hong Kong Special Administrative Region 999077, China; Mental Health Center, West China Hospital, Sichuan University, Chengdu 610041, China; The Province and Ministry Co-Sponsored Collaborative Innovation Center for Medical Epigenetics, Tianjin Medical University, Tianjin 300070, China; Stanley Center for Psychiatric Research, Broad Institute of MIT and Harvard, Cambridge, MA 02142, USA; Analytic and Translational Genetics Unit, Massachusetts General Hospital, Boston, MA 02114, USA; Department of Medicine, Harvard Medical School, Boston, MA 02115, USA; Zhongshan School of Medicine, Sun Yat-sen University, Guangzhou 510080, China; Key Laboratory of Tropical Disease Control (Sun Yat-sen University), Ministry of Education, Sun Yat-sen University, Guangzhou 510080, China; Center for Precision Medicine, Sun Yat-sen University, Guangzhou 510080, China

**Keywords:** Genetic interaction, Rare variant interaction burden, Truncated negative-binomial regression, Gene network, Complex disease

## Abstract

Genetic interactions play a crucial role in elucidating the susceptibility and etiology of complex multifactorial diseases. Despite significant efforts to identify disease-associated nonlinear effects in genome-wide association studies, efficient methods for detecting the epistatic impact of rare variants remain lacking. In this study, we propose iRUNNER, a novel and powerful mutation burden test focused on analyzing the interaction effects of rare variants on a binary trait. In contrast to conventional association tests that compare cases with controls, iRUNNER evaluates the relative enrichment of rare variant interaction burden of pairwise genes in patients against its baseline, estimated by a recursive truncated negative-binomial regression model that leverages multiple genomic features from public databases. Extensive simulations demonstrate that iRUNNER outperforms existing epistasis tests in statistical power and maintains reasonable type I error rates even when population stratification exists in control samples. When applied to real datasets from five complex diseases, iRUNNER yielded substantial gains in gene–gene interaction detection. Notably, the majority of these signals were missed by alternative methods, especially in small- to medium-sized samples. Furthermore, we found that these identified gene pairs of each trait can form interconnected networks, which may provide valuable insights into the underlying molecular mechanisms. We have implemented iRUNNER as a module in our integrative platform KGGSeq (http://pmglab.top/kggseq/) that enables rapid testing of pairwise interactions among all possible non-synonymous rare coding variants within hours.

## Graphical abstract

**Figure:**
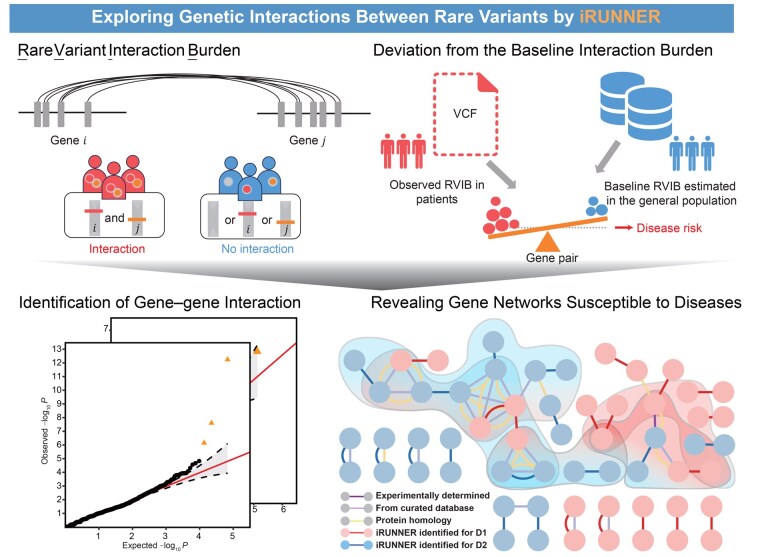

## Introduction

Complex human diseases are often influenced by multiple genes and their interactions. Despite advances in genome-wide association studies (GWASs), the genetic basis of these diseases remains largely unexplained, leading to the issue of “missing heritability” [1,2]. Genetic interactions, known as epistasis or gene–gene interaction (GGI), which refer to the deviation from the additive effects of two or more genetic variants on a phenotype, are thought to be a potential source of the underlying heritability [3,4]. The crucial role of epistasis in genetic architecture and disease susceptibility has been revealed in many complex human diseases, such as Alzheimer’s disease (AD) [5], schizophrenia [6], and inflammatory bowel disease [7], providing valuable insights into their etiology and facilitating the identification of potential drug targets. However, efficient methods for epistasis identification remain largely underdeveloped, particularly for the interactions among rare variants [*i.e.*, genetic variants with minor allele frequencies (MAFs) < 1%].

Methods for interaction analysis were originally explored at the single-nucleotide polymorphism (SNP) level, including parametric statistical methods, data mining, and machine-learning techniques. Logistic regression is a widely used parametric method for exhaustive searches of genetic interactions, as exemplified by the “--epistasis” option in PLINK (termed “PlinkEpi”) [8]. PLINK also provides an approximate but faster alternative test based on Z-scores, termed “FastEpistasis”, which tests the difference in odds ratios (ORs) between cases and controls. BOOST [9] accelerates analysis using the Boolean representation of genotype data for fast, bitwise, two-stage interaction tests. Despite differences in statistical models and assumptions, these methods essentially rely heavily on contingency table construction. For rare variant interactions, low MAFs lead to sparse contingency tables, which limit their detection power. An alternative class of detection strategies is offered by data-mining methods, exemplified by multifactor dimensionality reduction (MDR) [10]. The core principle of MDR is to collapse high-dimensional multi-locus genotype combinations into low-dimensional high-risk or low-risk values, thereby bypassing the need for parameter estimation or predefined genetic models. This model-free flexibility has spurred the development of numerous extensions, such as generalized MDR [11], odds ratio-based MDR [12], SVM-MDR [13], and spatial rank-based MDR [14]. However, MDR remains a brute-force search algorithm that becomes computationally prohibitive when analyzing a large number of SNPs, thereby limiting its scalability for genome-wide analyses [15]. With the rise of artificial intelligence, numerous machine-learning methods have been developed to detect combinations of variants associated with a phenotype, *e.g.*, Bayesian network-based methods such as BEAM [16], random forest approaches such as epiForest [17], and ant colony optimization algorithms like epiACO [18]. These methods can reveal associations of variants potentially involving epistasis, but many cannot distinguish whether the association is due to interactive or additive effects of multiple independent SNPs. Moreover, such methods designed to test for interactions within common variants are underpowered for analyzing rare variants unless sample sizes or effect sizes are extremely large.

To increase power, a typical approach is to take a gene or genome region as the basic unit of analysis, shifting the focus from SNP–SNP epistasis tests to GGI studies. This strategy has been employed by several interaction methods designed for low-frequency or rare variants, such as FRG [19], FLR [20], IGOF [21], and GxGrare [22]. Such methods aggregate information from multiple variants and collectively test interactions between variant pairs within two genes, evaluating statistical significance by comparing the genotype distributions of the gene pairs between cases and controls. To quantify these distributional differences, strategies such as functional regression [19], OR [20], Pearson’s goodness-of-fit statistic [21], or MDR [22] are employed, with *P* values calculated based on specific distributions (*i.e.*, chi-square distribution) or permutations. Recently, FastKAST [23] and QuadKAST [24] further enlarged the test unit into “sets” and then tested the aggregated linear and nonlinear effects of all possible variants within each set with a kernel function. Despite the improved power compared to SNP-based epistasis tests, existing GGI tests for rare variants still exhibit limitations in exome-wide or genome-wide analyses due to insufficient sample sizes and excessive computing demands. Therefore, there is an urgent need to develop scalable and powerful tests for robust detection of genetic interactions among rare variants.

The development of reference population databases [25–27] has facilitated the creation of diverse resources for variant and gene interpretation, opening new opportunities to map genotype–phenotype associations. Incorporating functional annotations, such as conservation scores [28,29] and pathogenic predictions [30–32], has been widely accepted and successfully used to boost the power of rare variant association studies [33–36]. Recently, the RUNNER method [37] further advanced rare variant burden testing by accurately modeling the expected mutation burden across genes through regression on multiple genomic features available from public databases. Compared to conventional association tests (*i.e.*, CMC [38], SKAT [34], and KBAC [39]), RUNNER demonstrated superior performance in studies with small or medium sample sizes due to its unique testing strategy: comparing a gene’s observed rare mutation burden in patients against its baseline expectation in the general population. By leveraging external databases to address the prevalent challenge of inadequate sample sizes, RUNNER holds substantial promise for enhancing statistical power in exploring rare-variant-based GGIs.

Another major challenge in genome-wide analyses of GGI is the exponential growth in the number of interactions that need to be tested as the number of genes increases. For example, an exhaustive search for pairwise interactions among n genes requires testing n(n−1)/2 combinations. This issue becomes even more challenging when high-order interactions are considered, creating a heavy computational burden and statistical challenges for multiple testing corrections [40]. Focusing on specific subsets of interactions based on prior knowledge or functional relevance can effectively reduce the testing space. In this context, our previous work, the digenic interaction effect predictor (DIEP) [41], provides a valuable resource for researchers to identify pathogenic gene pairs with potential digenic interaction effects. For instance, by considering gene pairs with a DIEP prediction score greater than 0.5 in human protein-coding genes (approximately 20,000 genes), pairwise interactions can be reduced from around 400 million in an exhaustive search to nearly 4 million (1%). Applying a more stringent threshold can further narrow the search space, thus substantially reducing both computational demand and the burden of multiple testing corrections.

In this study, we propose a novel GGI test, iRUNNER, as an extension of RUNNER, designed to detect the interaction effects of multiple rare variants between gene pairs on a binary trait. The proposed method models the baseline mutation burden across pairwise genes by leveraging genomic features from the gnomAD database [25]. Then, the enrichment of mutation burden in patients relative to the estimated baseline burden is evaluated to identify GGIs susceptible to complex diseases. We performed extensive simulations to demonstrate that iRUNNER maintains reasonable false positive rates and achieves greater power in detecting rare variant interactions compared to existing methods across a range of epistatic disease models. We also applied iRUNNER to five complex diseases using in-house datasets and whole-exome sequencing (WES) data from the UK Biobank (UKBB), uncovering multiple promising signals that were missed by alternative methods.

## Method

### Notation and model

Suppose there are K affected individuals sequenced in the study. Consider two genes, i and j, and each gene is a collection of $V_{i}$ and $V_{j}$ rare variants, respectively. Let $g_{i,p,k}=$ 0, 1, or 2 represent the copy number of the minor allele for variant p in gene i carried by the k-th case subject ($p\in V_{i},\;k=1,2,\ldots ,K$). A similar representation $g_{j,q,k}$ is used for variant q in gene j ($q\in V_{j}$). Here, we used rare variant interaction burden (RVIB) to measure the degree of rare variants with potential interaction effects on a pair of genes. A basic RVIB of the gene pair (i,j) observed in cases, $y_{ij}$, can be measured based on genotype functions as follows:


(1) yij=∑k=1Kmax⁡{gi,p,k⋅gj,q,k∣p∈Vi, q∈Vj}

In the calculation, only variant pairs with at least one copy of the minor allele present at both loci are considered potentially interacting. This assumption is consistent with the two-locus interaction model [42]. RVIB can be further improved by incorporating functional annotations of variants in a gene. Assume a variant *v* of gene *G* has a score, $s_{v,G}\in [0,1]$, indicating its pathogenic potential. Given a score bin length b∈[0,1], $s_{v,G}$ can be converted to an integer functional weight by the ceiling function as: $w_{v,G}=[s_{v,G}/b]$. Therefore, the functional weighted RVIB of the gene pair(i,j) is calculated as:


(2) Yij=∑k=1Kmax⁡{wi,pgi,p,k⋅wj,qgj,q,k∣p∈Vi, q∈Vj},

where $w_{i,p}$ and $w_{j,q}$ are the integer functional weights of rare variant p on gene i and variant q on gene j, respectively. In this study, functional weights were assigned based on integrated deleterious scores predicted using the logistic regression model embedded in the KGGSeq software [43]. For downstream analyses, unless otherwise specified, we used the functional weighted $Y_{ij}$ to calculate rare variant interaction burden for tested gene pairs. By analyzing the distribution characteristics in real data (Figure S1), we assumed that the RVIB statistic $Y_{ij}$ is asymptotically distributed as a truncated negative-binomial (TNB) distribution:


(3) Yij∼TNB(μij,θ,t),Yij=t+1,t+2,……,

where *µ*${\;}_{ij}$ is the expected baseline RVIB of the gene pair (i,j), and *θ* is a dispersion parameter. A notable distinction from the classical negative-binomial distribution is that a flexible data-dependent truncation point t is introduced in the TNB distribution to restrict the range of values of $Y_{ij}$ for a better fit of the likelihood function. Details about the TNB distribution are described in File S1.

By default, iRUNNER uses prediction scores from DIEP [41] to narrow down all pairwise gene interactions to a subset of gene pairs with potential pathogenic interaction effects. The DIEP scores were precalculated for all protein-coding gene pairs across the human genome, and 3,920,174 gene pairs were predicted with a DIEP score over 0.5. The distribution of DIEP scores ([0, 1]) can be seen in Figure S2.

### iRUNNER estimates the expected RVIB based on multiple genomic features

Based on the TNB distribution, iRUNNER constructs a generalized linear regression model to estimate the expected RVIB in the general population and test the enrichment of rare variant interactions at gene pairs in patients. In the present study, six genomic features potentially impacting the number of rare mutations per gene were incorporated in the regression model. These features were adapted from our previous work, RUNNER [37], and represent the optimally performing subset selected from eight candidate predictors through combinatorial testing. For a single gene, these features include the coding region (CDS) length ($X_{1}$), the accumulated MAF ($X_{2}$), the product of CDS length and MAF ($X_{3}$), the observed/expected ratios for missense (oe_mis,$\;X_{4}$) and loss-of-function variations (oe_lof, $X_{5}$), and the GC content ($X_{6}$). Values for these six features can be obtained or calculated from gnomAD [25] (https://gnomad.broadinstitute.org/data), a detailed calculation for the $X_{2}$ is given in File S1. When analyzing a gene pair (i,j), the value for each feature is calculated as the product of the corresponding feature values of gene i and gene j. Additionally, a similar rare variant interaction burden of the gene pair (i,j) observed in controls can be calculated and included in the regression model as the seventh predictor $X_{7,ij}$. However, if the number of control subjects is limited (*e.g.*, < 50) in the studied sample, $X_{7,ij}$ will not be considered in the regression model, so we added an asterisk to this term in the equation below. Under a link function, the expected RVIB of gene pair (i,j) in the studied sample can be estimated as:


(4) ln⁡(µij)=β0+∑l=16βlXl,iXl,j+β7X7,ij, *

where $\beta_{0}$ is an intercept, and $\beta_{1}$ to$\;\beta_{7}$ are the regression coefficients for $X_{1}$ to $X_{7}$, respectively. Since the vast majority of rare variant interactions between genes are not associated with disease status, we can simply let *µ*_*ij*_  $=Y_{ij}$ in estimating the regression coefficients. Given a specific score bin length b for integerizing the function weight and a truncation point t, then the regression coefficients ($\beta_{0},\;\beta_{1},\cdot \cdot \cdot ,\;\beta_{6},\;\beta_{7}^{*})$ and dispersion parameter (*θ*) of the TNB distribution model can be estimated by maximum likelihood with a Quasi-Newton method. With the fitted coefficients, the deviance residuals are calculated and standardized as ${\overset{´}{d}}_{ij}$ (File S1). A large ${\overset{´}{d}}_{ij}$ indicates the gene pair (i,j)’s observed RVIB in patients is greater than its expected RVIB in the general population. The ${\overset{´}{d}}_{ij}$ can be approximated using a standard normal distribution N(0, 1), and its *P* value is calculated as $1-\Phi ({\overset{´}{d}}_{ij})$ where Φ is the cumulative distribution function of the standard normal distribution.

In practice, we also propose to employ a recursive regression procedure to purify the model for the background gene pairs. First, we fitted the model with all the gene pairs and identified the gene pairs with a slightly looser significant threshold [*e.g.*, Benjamin–Hochberg false discovery rate (BHFDR) *q* value ≤ 0.1]. We removed these gene pairs and refitted the model with the remaining ones. This process was repeated until no gene pair had a large deviance residual or the significant gene pairs became stable. Then, the resulting set of gene pairs formed the background gene pairs. During each iteration, the regression coefficients were re-estimated using maximum likelihood with a Quasi–Newton method under the TNB distribution. Then, we used the estimated coefficients to calculate *P* values for all the gene pairs, which were used to identify gene pairs for removal in the next iteration. This procedure allowed us to model the background gene pairs while minimizing the influence of true disease-associated gene pairs with excessive rare variant interaction burden on the coefficient’s estimation.

The optimal values for score bin length b and truncation point t were explored under a grid-search procedure. We tested values of b ranging from 0.025 to 0.5 with an interval of 0.025, and values of t ranging from 0 to 2 with a break of 1. The optimal combination of b and t was determined as the value that minimizes the mean log fold change (MLFC) [44], which measures the departure of *P* values of background gene pairs from the uniform distribution, and maximizes the number of significant gene pairs according to a balanced ranking. The recursive procedure under the specific combination of b and t will be stopped if the number of gene pairs used for regression fitting is less than 5000 to avoid overfitting. Finally, iRUNNER calculated *P* values for all tested gene pairs based on the stable regression model using the optimal combination of b and t, and provided the results to the users.

#### Experiments on simulation data

##### Datasets used in simulations

We used a semi-simulation procedure to evaluate the performance of iRUNNER. Whole-genome sequencing data from the Singapore 10K genome project (SG10K) [45] were used for type I error and power simulations. The SG10K dataset comprises 4810 individuals from Singapore, available through the European Genome-Phenome Archive (EGA, https://ega-archive.org) under accession number EGAS00001003875. After removing samples related within three degrees of kinship using KING [46] (File S1), 4016 unrelated individuals remained. To avoid potential confounding from pre-existing interaction signals, we further excluded variants in 19 genes that repeatedly exhibited significant interactions within this subset (Table S1). For population-stratification analyses, five ancestry panels of the 1000 Genomes Project (1KGP, ftp://ftp.1000genomes.ebi.ac.uk/vol1/ftp/release/20130502/) [27] were used for the simulation, including African (AFR, n=661), American (AMR, n=347), East Asian (EAS, n=504), European (EUR, n=503), and South Asian (SAS, n=489).

#### Type I error simulations

We first performed simulation studies under the null hypothesis (no true associations) by randomly sampling with different sizes (n=1000, 2000, 3000, and 4000) without replacement from the 4016 unrelated SG10K individuals; half of them were labelled as pseudo-cases, the remainder served as controls. Rare variants with an MAF of less than 1% in the East Asian panel of gnomAD exomes and gnomAD genomes were included in the analysis. We also carried out simulation studies under varied MAF cutoffs (0.1%, 0.5%, 1%, and 5%) to examine their impact on type I error rates with a fixed sample size of 4000. iRUNNER was performed on gene pairs with DIEP scores over 0.9. Average type I error rates were calculated based on 100 replicates at significance levels *α*  $=10^{-3},10^{-4}$, and $10^{-5}$. The distribution of *P* values was visualized using the quantile–quantile (QQ) plot of ${-\log}_{10}\;P$ under the uniform distribution U[0,1] and the MLFC was used to measure deviations from the uniform distribution.

To evaluate the effects of population stratification on the type I error rates, we utilized subjects from the 1KGP dataset to generate samples with ancestrally mixed controls. In each sample, two-thirds of individuals were randomly selected from a specific ancestry panel (*e.g.*, EAS) to serve as pseudo-cases, while the remaining one-third, along with an equal number from another ancestry panel (*e.g.*, AFR), formed the controls. Overall, we generated 20 such samples by varying the panel combinations. iRUNNER was applied to rare variants with an MAF of less than 1% in the gnomAD panel that matched the ancestry of the cases.

#### Power simulations

Empirical power was assessed under the alternative hypothesis by inserting causal variants into the SG10K genomes. Unrelated subjects were randomly assigned as pseudo-cases or controls, and the *PARK7*–*PINK1* interaction was designated as the target gene pair. *PARK7* and *PINK1* form a well-documented interaction whose joint dysfunction is causal for autosomal recessive Parkinson’s disease [47]. Variants in these genes were pre-removed to avoid background signals. *P* values obtained by iRUNNER were compared with those from seven alternative methods, including three SNP–SNP pairwise epistasis tests implemented in PLINK v1.90b6.12 (PlinkEpi, FastEpistasis, and BOOST [9]), two kernel-based approaches that test for nonlinear effects of variant sets (FastKAST [23] and QuadKAST [24]), and two GGI methods designed for low-frequency variants (FRG [19] and GxGrare [22]). As some tools do not aggregate mutations within a gene, we assumed that only one pair of rare variants associated with susceptibility exists within this target gene pair (Table S2).

Causal genotypes were generated under three built-in two-locus interaction models: the threshold model [42], the multiplicative model [42], and the classic epistasis model [9]. **Table 1** summarizes these models, showing the odds of diseases for each genotype combination at two loci, A (disease risk allele a) and B (disease risk allele b). The symbols *α* and *θ* represent the baseline risk and effect size, respectively. Specifically, the threshold model assumes that the odds have a baseline value unless both loci have at least one disease-associated allele, but additional copies of these alleles do not increase the risk further. The multiplicative model specifies that each additional copy of the disease-associated allele at loci A and B further increases the odds in a multiplicative fashion. The classic epistasis model involves an interaction where one allelic effect is blocked by another allele at a different locus. Both loci have the same effect size. For a specific model, the *α* and *θ* can be calculated based on the pre-specified disease prevalence Pr(*D*), and the MAF and genotype OR of the assumed disease-associated variants [48] (see details in File S1).

**Table 1:** Odds of the three epistatic disease models

Disease model	Locus B	Locus A		
		AA	Aa	aa
The threshold model	BB	α	α	α
	Bb	α	α (1+θ)	α (1+θ)
	bb	α	α (1+θ)	α (1+θ)
The multiplicative model	BB	α	α	α
	Bb	α	α (1+θ)	$\alpha \;{\left(1+\theta \right)}^{2}$
	bb	α	$\alpha \;{\left(1+\theta \right)}^{2}$	$\alpha \;{\left(1+\theta \right)}^{4}$
The classic epistasis model	BB	α	α	α (1+4θ)
	Bb	α	α (1+2θ)	α
	bb	α (1+4θ)	α	α

*Note*: α, baseline risk; θ, effect size.

In this study, the Pr(*D*) was fixed at 1% across all epistasis scenarios. We began simulations by assigning each causal variant a MAF of 1%, then explored a grid of ORs (5, 10, 15, and 20) crossed with sample sizes ranging from 1000, 2000, 3000, to 4000 individuals under balanced case-control designs. To evaluate the effects of MAF on power, we varied MAFs (1%, 0.25%, 0.5%, 1%, and 2%) while maintaining a constant OR of 20 and a sample size of 4000 (2000 cases and 2000 controls). For each scenario, 100 replicate datasets were generated. Statistical power was defined as the proportion of replicates in which the *PARK7–PINK1* pair was detected as a significant signal. The 95% confidence interval (CI) was calculated using the Clopper–Pearson exact method for binomial proportions.

### Application to real data

We then applied iRUNNER to five real high-throughput sequencing (HTS) datasets, including two in-house complex disease datasets: AD [49] and Hirschsprung’s disease (HSCR) [50], and three datasets from the UK Biobank (UKBB) within the WES data [51]: ulcerative colitis (UC), Crohn’s disease (CD), and type 2 diabetes (T2D). Sample information of the five datasets is summarized in Table S3. All sequenced samples in AD and HSCR studies were obtained with Institutional Review Board approval in Hong Kong or mainland China. Human genetics data from the UKBB were accessed via collaboration with Application No. 86920.

All analyses for detecting susceptibility GGIs between rare variants from the HTS data were conducted in our KGGSeq platform (v1.2, http://pmglab.top/kggseq/). Quality control (QC) was performed in the following steps. We first removed the variants with the Hardy–Weinberg equilibrium *P* value ≤ $1\times 10^{-5}$ and alleles of more than four types. Second, genotypes with read depth less than 8 or a genotyping quality score (Phred Quality Score) < 20 were changed to a no-call. Finally, variants with more than 20% missing genotypes were excluded. After QC, we performed iRUNNER analysis of rare non-synonymous variants (missense, start-loss, stop-loss, stop-gain, splicing, frameshift, and non-frameshift variants that were annotated by RefGene [52] and GENCODE [53]) for protein-coding gene pairs with DIEP scores over 0.8. Eight genes (*TTN*, *MUC16*, *OBSCN*, *NEB*, *MUC19*, *MUC4*, *DST*, and *DNAH14*) with extremely long CDS lengths were excluded from the analysis. Table S3 also provides information on the number of rare variants and gene pairs tested in each dataset.

We further queried the STRING database [54] (https://string-db.org/) to identify published information on protein–protein interactions (PPIs). PPIs of five categories (including experimentally determined, curated databases, gene co-occurrence, co-expression, and protein-homology) with confidence levels exceeding 0.5 were used for visualization. GGIs were visualized using Cytoscape software [55]. To characterize the biological functions of iRUNNER-identified genes, Gene Ontology (GO) [56] enrichment analysis was performed using the clusterProfiler package (v4.2.2) [57] in R. The enrichment significance *P* values were calculated via hypergeometric tests.

## Results

### Overview of methods

**Figure 1:**
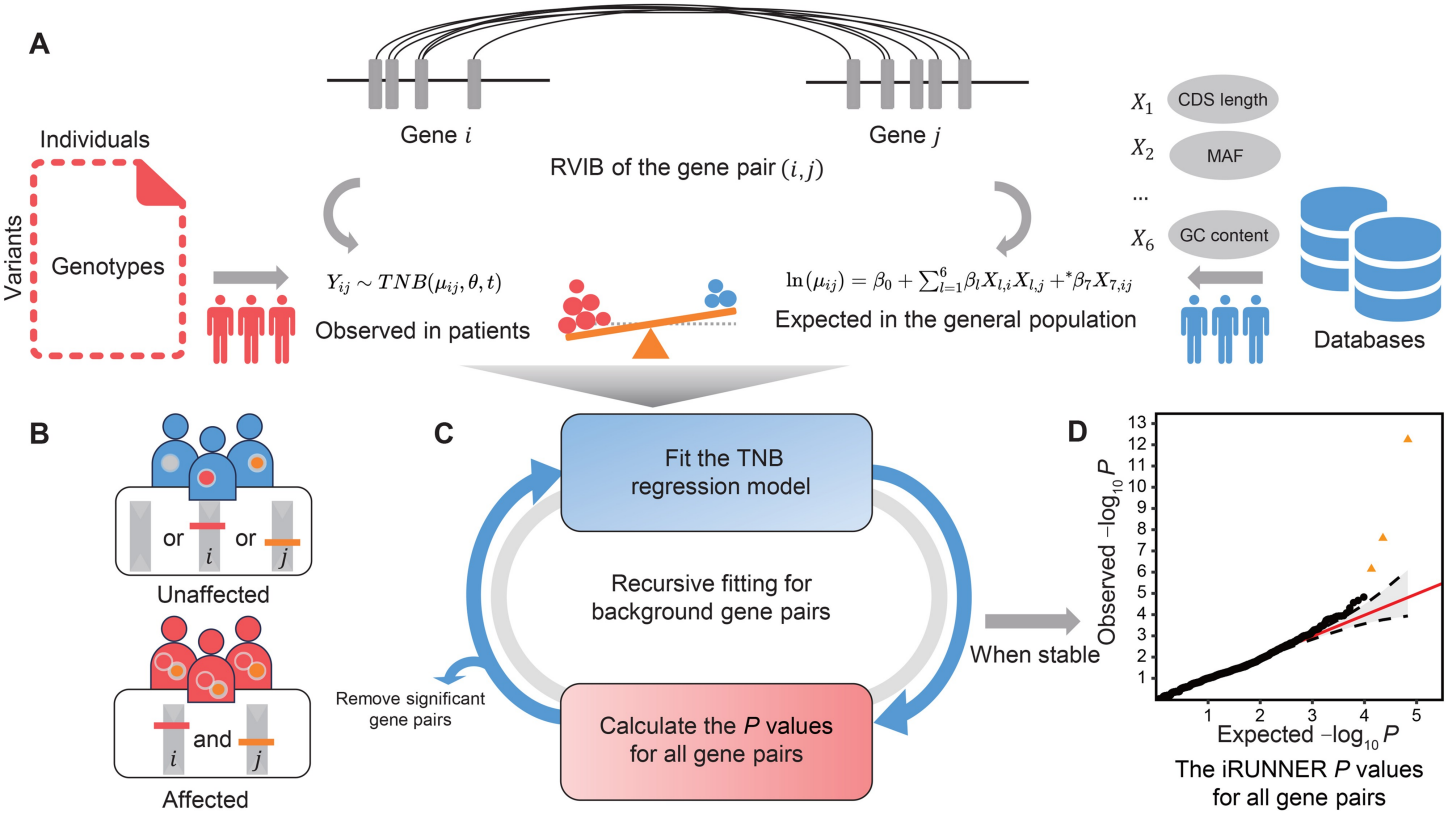
iRUNNER framework overview **A**. iRUNNER evaluates the enrichment of RVIB in patients compared to its baseline expectation in the general population. For each pre-tested gene pair, iRUNNER calculates the observed RVIB in cases based on input genotype data uploaded from users (left part) and estimates the expected RVIB by regressing multiple genomic features derived from external public annotations (right part). **B**. In the calculation of RVIB, only those gene pairs that are simultaneously mutated in a patient are considered as having the potential to increase disease risk. **C**. Recursive fitting of the TNB regression model to obtain a stable model constructed on background gene pairs. **D**. The iRUNNER *P* values for all tested gene pairs are calculated and reported based on the stable regression model. RVIB, rare variant interaction burden; TNB, truncated negative-binomial; CDS, coding sequence; MAF, minor allele frequency.

iRUNNER is an innovative statistical method for analyzing GGIs between rare variants. It evaluates whether interactions between rare variants in pairs of genes are significantly enriched in disease risk by comparing the observed rare variant interaction burden in patients to its baseline expectation in the general population. There are three main steps in the iRUNNER framework: (1) calculating the RVIB in cases for pre-tested gene pairs and preparing predictor values based on public annotation databases; (2) recursively fitting the truncated negative-binomial regression model on background gene pairs, and (3) testing for significance of the deviation from the baseline RVIB for all tested gene pairs based on the fitted regression model (**Figure 1**).

A key technical challenge for iRUNNER is accurately estimating the baseline rare variant interaction burden for gene pairs. To address this, we developed the RVIB statistic to measure the genetic burden of potentially harmful rare variant interactions within gene pairs among patients. For each patient, only those gene pairs that are simultaneously mutated are considered as having the potential to increase disease risk (Figure 1B). Then, we utilized a truncated negative-binomial regression model to estimate the baseline expectation based on multiple genomic features derived from public annotation databases (Figure 1A; Method). This model offers advantages over the standard negative-binomial model by incorporating a data-driven truncation point, t, which accommodates the inflated number of gene pairs with low interaction burdens. Additionally, iRUNNER employs a recursive regression procedure, similar to outlier removal in regression analysis, to model background gene pairs and generate baseline expectations under the null hypothesis (Figure 1C). This approach helps establish a reliable baseline expectation for RVIB. To assess the significance of GGIs, iRUNNER compares the RVIB observed in patients with its estimated baseline expectation and calculates a corresponding *P* value for each tested gene pair (Figure 1A and D).

### Evaluation of type I error rates and robustness of iRUNNER

We first assessed the performance of iRUNNER in controlling type I error using randomly sampled datasets from unrelated SG10K individuals under the null hypothesis. As shown in **Table 2**, empirical type I error rates were properly controlled across all significance levels (*α*  $=10^{-3}$, $10^{-4}$, and $10^{-5}$), sample sizes (n=1000, 2000, 3000, and 4000), and MAF cutoffs (MAF = 0.1%, 0.5%, 1%, and 5%). The distribution of *P* values for tested gene pairs closely followed the uniform distribution U[0,1] (Figure S3), even at smaller sample sizes. We further quantified the departure of *P* values from U[0,1] using the MLFC. Across all tested conditions, the MLFC values were consistently stable and moderate (< 0.2; Table 2).

**Table 2:** Empirical type I error rates of iRUNNER for testing GGI between rare variants

Sample size	MAF cutoff	Alpha			Number of tested gene pairs	MLFC
		1.0E−03	1.0E−04	1.0E−05		
1000	1%	4.144E−04	4.726E−05	7.111E−06	16848 ± 606	0.161 ± 0.015
2000	1%	5.325E−04	6.435E−05	7.483E−06	20216 ± 548	0.143 ± 0.019
3000	1%	5.921E−04	6.928E−05	9.818E−06	22403 ± 524	0.132 ± 0.018
4000	1%	6.203E−04	6.971E−05	5.094E−06	23540 ± 411	0.129 ± 0.019
4000	0.1%	5.813E−04	7.126E−05	8.037E−06	19933 ± 483	0.144 ± 0.018
4000	0.5%	6.177E−04	7.003E−05	3.836E−06	23428 ± 415	0.130 ± 0.018
4000	5%	6.394E−04	7.235E−05	8.082E−06	23402 ± 1590	0.128 ± 0.018

*Note*: iRUNNER was applied to samples with different sample sizes (*n* = 1000, 2000, 3000, and 4000), and tested GGIs of rare variants under different MAF cutoffs (0.1%, 0.5%, 1%, and 5%). The semi-simulation procedure produced the samples based on whole-genome sequencing data from unrelated SG10K subjects under a balanced case-control design. Each setting repeats 100 times. MAF, minor allele frequency; MLFC, the mean log_2_ fold change; GGI, gene–gene interaction.

We then investigated the impact of population stratification using 20 samples with ancestral mixed controls simulated based on 1KGP data. Overall, iRUNNER showed no systematic inflation of spurious associations, even when up to 50% of controls were stratified, as evidenced by the QQ plots in Figure S4. The MLFC values (Table S4) remained comparable to those observed in SG10K simulations. In some samples, a few gene pairs reached study-wide significance (BHFDR *q* value < 0.05; Figure S4). For example, the *CREB1–SPP1* pair was consistently identified across resampled admixed controls for pseudo-cases in AMR, suggesting that the elevated interaction burden originates from the case set, rather than random noise. Taken together, these findings demonstrate that iRUNNER can effectively control for the impacts of population stratification that exist in controls and ensure the reliability of rare variant interaction analysis.

### Power comparison of iRUNNER with existing methods

**Figure 2:**
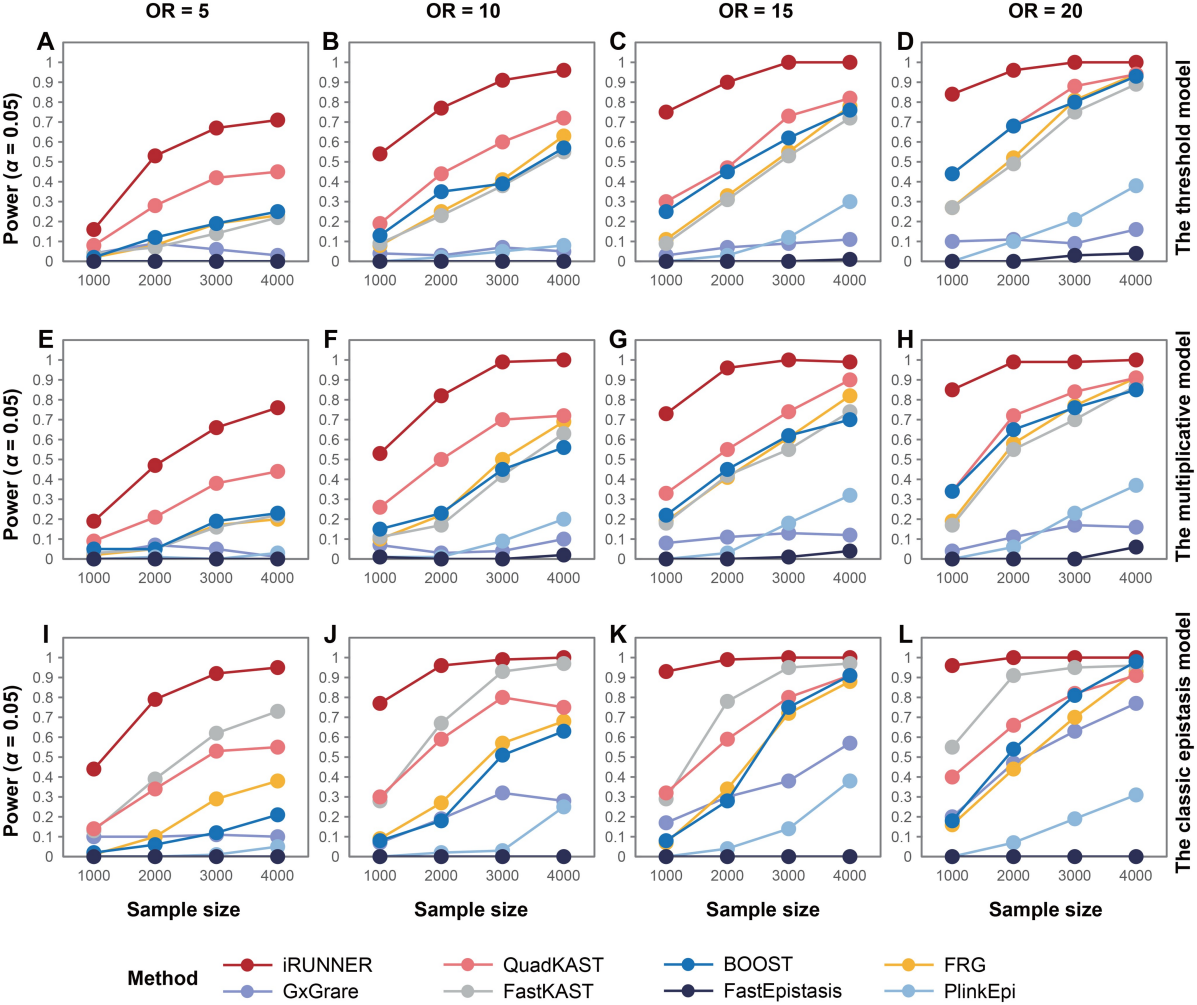
Power curves of iRUNNER and competing methods The power of iRUNNER and seven alternative methods was tested under three established disease models: the threshold model (**A**.–**D**.), the multiplicative model (**E**.–**H**.), and the classic epistasis model (**I**.–**L**.). For each model, panels from left to right represent scenarios where the genotype OR of causal variants is 5 (A, E, I), 10 (B, F, J), 15 (C, G, K), and 20 (D, H, L). In all scenarios, the MAF was fixed at 1% and the disease prevalence at 0.01. The power of eight methods was tested at sample sizes of 1000, 2000, 3000, and 4000. Samples were generated through a semi-simulation procedure based on whole-genome sequencing data from unrelated SG10K subjects. Power was estimated as the proportion of *P* values < 0.05 among 100 replicates. OR, odds ratio.

Next, we compared iRUNNER with seven existing methods to assess its effectiveness in detecting causal rare variant interactions between two genes. The diseased samples were simulated by randomly inserting risk alleles into SG10K genomes under three epistasis models: the threshold model, the multiplicative model, and the classic epistasis model. We first evaluate power with increasing sample sizes, with causal variants having varying genotype ORs while maintaining a fixed MAF of 1%, the threshold commonly used to define rare variants. As shown in **Figure 2**, our proposed iRUNNER demonstrated superior performance over alternative methods at the significance level of *α*  =0.05 across all simulated conditions. For example, under the threshold model, iRUNNER achieved a power of 91% (95% CI: 83.60%–95.80%; Table S5) with OR = 10 and sample size *n* = 3000, outperforming the best alternative method (QuadKAST: 60%; 95% CI: 49.72%–69.67%) by 31%. Increasing effect size and sample size further enhanced the power of iRUNNER, as well as QuadKAST, BOOST, FRG, and FastKAST, while small improvements were observed for GxGrare, PlinkEpi, and FastEpistasis (Figure 2A–D). Under the multiplicative (Figure 2E–H) and the classic epistasis models (Figure 2I–L), iRUNNER consistently exhibited the highest power, with power increasing as the sample size and effect size of causal variants increased.

We then explored scenarios with causal variants having lower MAFs (0.1%, 0.25%, 0.5%) or higher MAFs (2%), fixing OR = 20 and *n* = 4000. As shown in Figure S5, iRUNNER still outperformed other methods in detecting causal interactions across all three epistasis models. Notably, when the causal variants had a lower MAF of 0.5%, iRUNNER achieved much higher power than other methods at *α*  =0.05. For instance, under the classic epistasis model with an OR of 10, iRUNNER achieved 83% power (95% CI: 74.18%–89.77%), far exceeding the best alternative, which was only 43% (FastKAST, 95% CI: 33.14%–53.29%). When the MAF decreased to 0.1%, all methods showed low power, but the power of iRUNNER substantially increased as the MAF rose to 0.25% and the OR increased from 5 to 20. When the MAF in the population reached 2%, the causal variants in the simulated cases could be considered common variants (MAF > 5%) under the assumed ORs. In this scenario, we observed a significant increase in power for PlinkEpi, FastEpistasis, and GxGrare, but iRUNNER still showed the highest power of 100% (95% CI: 96.38%–100.00%).

To evaluate the stability of power estimation, we expanded the simulations in Figure 2B from 100 to 1000 replicates and further conducted 100 bootstraps (100 replicates each). The resulting average power ± SD exhibited little fluctuation, confirming the reliability of the estimates and iRUNNER’s consistent superiority over alternative methods (Table S6). We also assessed all methods at a stricter significance level of *α*  $=1\times 10^{-6}$. As shown in the power curves in Figures S6 and S7, this stringent threshold did not significantly affect power patterns for iRUNNER, whereas the alternative methods nearly lost their ability to detect causal interactions unless the causal variants had relatively high MAFs.

### Identification of GGIs susceptible to complex diseases by iRUNNER

We further applied iRUNNER to real high-throughput sequencing datasets to identify susceptibility GGIs among rare variants. Five complex disease datasets, differing in sample size, population ancestry, and genetic structure, were used as examples for application.

#### Alzheimer’s disease

**Figure 3:**
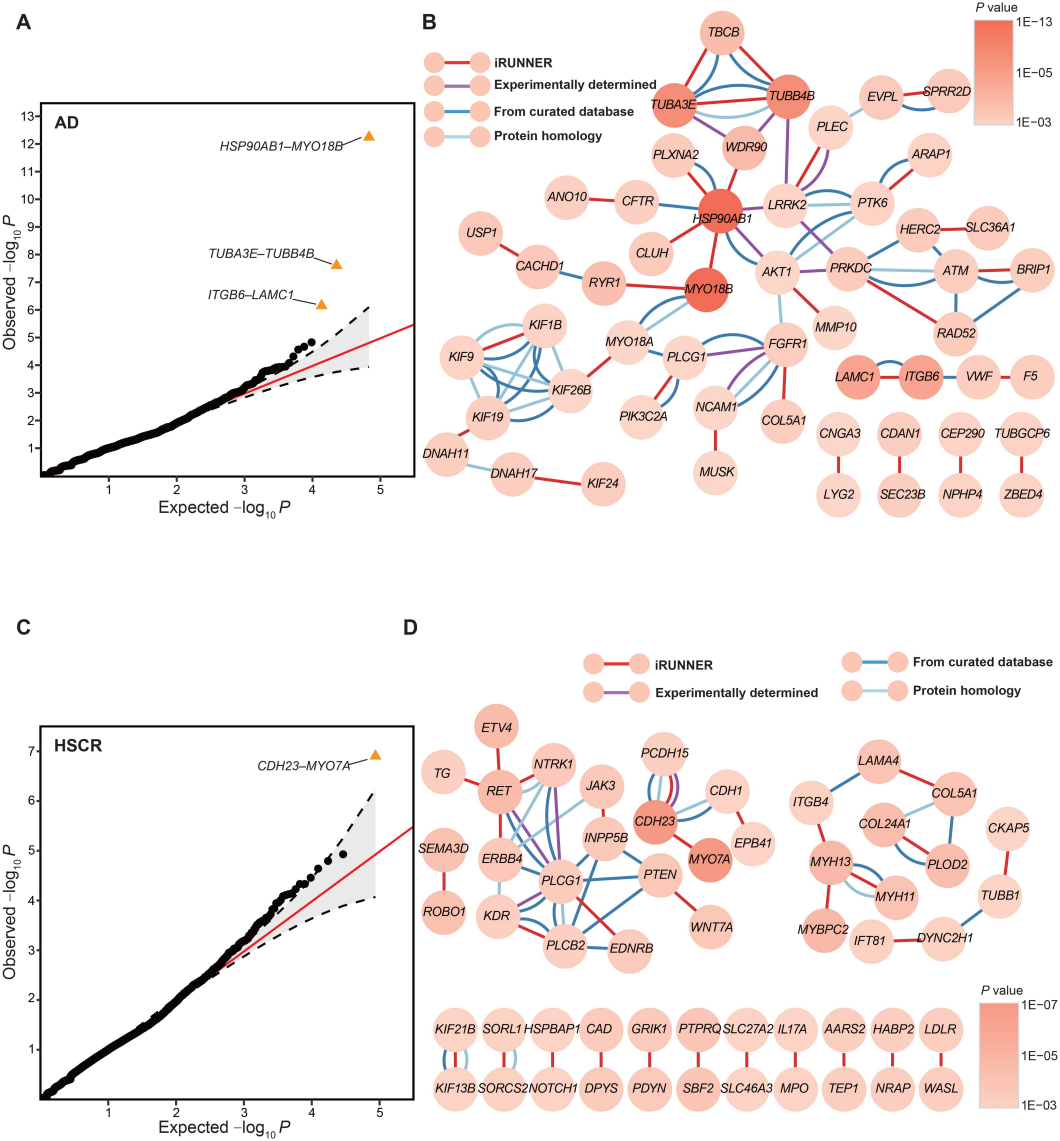
Discovery of GGIs associated with AD and HSCR by iRUNNER **A**. The QQ plot of *P* values generated by iRUNNER in AD (246 cases, 172 controls). The orange triangles indicate significant GGIs (BHFDR *q* < 0.05). **B**. The gene network formed by the top 30 interactions associated with AD identified by iRUNNER. The darker orange nodes correspond to genes with more significant iRUNNER *P* values. The red edges indicate interactions identified by iRUNNER. Other colored edges originate from interactions with confidence scores ≥ 0.5 in each STRING category (purple line for experimentally determined interactions; dark blue line for interactions from curated databases; light blue line for protein homology), and text-mining connections are not considered. **C**. The QQ plot of *P* values generated by iRUNNER in HSCR (443 cases, 493 controls). **D**. The gene network formed by the top 30 interactions associated with HSCR identified by iRUNNER. AD, Alzheimer’s disease; HSCR, Hirschsprung’s disease; BHFDR, Benjamin–Hochberg false discovery rate; GGI, gene–gene interaction.

AD is a neurodegenerative disorder primarily characterized by cognitive decline and memory loss. The AD dataset consists of 246 Hong Kong Chinese patients with ApoE ɛ4-negative AD and 172 ethnic-matched controls [49]. After quality control at variants and genotypes, a total of 53,769 rare variants in 14,599 genes were retained, and 52,892 pairs of protein-coding genes were included in the interaction analysis (summarized in Table S3). In such a small sample, iRUNNER still obtained well-calibrated *P* values, as shown in the QQ plot in **Figure 3**A.

The ten most significant GGIs identified by iRUNNER are detailed in **Table 3**. Remarkably, the most significant result was the interaction between *HSP90AB1* and *MYO18B* genes, yielding a *P* value of $5.23\times 10^{-13}$, which also reached study-wide significance (BHFDR *q* value $=1.77\times 10^{-8}$). *HSP90AB1*, a member of the Hsp90 family of chaperones, is known to participate in AD pathology by mediating the autophagic clearance of tau and amyloid-β (Aβ) aggregates [58]. The second significant GGI  was *TUBA3E–TUBB4B* ($P=2.29\times 10^{-8},q=3.88\times 10^{-4}$). Both *TUBA3E* and *TUBB4B* are members of the tubulin gene family, encoding α-tubulin and β-tubulin subunits, respectively. α- and β-tubulin are crucial for microtubule stabilization and have been linked to AD previously [59]. Additionally, we identified another tubulin-related gene, tubulin folding cofactor B (*TBCB*), with potential interactions with *TUBA3E* ($P=2.02\times 10^{-5}$, q=0.137) and *TUBB4B* ($P=4.56\times 10^{-5}$, q=0.221). *TBCB* is predicted to play a role in nervous system development and is highly expressed in the brain. The gene pair *ITGB6–LAMC1* also achieved study-wide significance ($P=6.50\times 10^{-7}$, $q=7.35\times 10^{-3}$). *ITGB6* encodes a subunit of the integrin, a family of cell-surface adhesion receptors that is likely to contribute to imbalanced synaptic function in AD [60]. *LAMC1* encodes a member of the laminins, an important extracellular matrix component. Studies have suggested that laminins interact with Aβ and contribute to the formation of amyloid plaques, a characteristic feature of AD brains [61]. By integrating known interactions documented in the STRING database, we observed that the top 30 interactions (involving 53 genes) identified by iRUNNER formed a tightly interconnected network (Figure 3B), which holds promising potential for studying the pathways contributing to AD susceptibility.

**Table 3:** Top 10 GGIs identified by iRUNNER and their corresponding *P* values in competing methods

Trait	Gene 1	Chr	Gene 2	Chr	*P* value				
					iRUNNER	GxGrare	PlinkEpi (min)	FastEpistasis (min)	BOOST (min)
AD	*HSP90AB1*	6	*MYO18B*	22	5.23E−13	0.1040	0.9890	0.1707	0.0133
	*TUBA3E*	2	*TUBB4B*	9	2.29E−08	0.2271	0.9884	0.2712	0.0129
	*ITGB6*	2	*LAMC1*	1	6.50E−07	0.5598	0.9960	0.3079	1.0000
	*HSP90AB1*	6	*WDR90*	16	1.40E−05	0.0027	/	0.2292	0.5298
	*TBCB*	19	*TUBA3E*	2	2.02E−05	0.6577	/	0.3479	1.0000
	*MYO18B*	22	*RYR1*	19	2.57E−05	0.3089	0.9876	0.3077	0.0217
	*TBCB*	19	*TUBB4B*	9	4.56E−05	0.1530	/	0.3827	1.0000
	*CACHD1*	1	*USP1*	1	7.86E−05	0.1204	/	0.3788	1.0000
	*EVPL*	17	*SPRR2D*	1	1.05E−04	0.0083	0.9915	0.1023	0.2650
	*PRKDC*	8	*RAD52*	12	1.09E−04	0.1943	/	0.2838	0.6650

*Note*: iRUNNER was performed on rare non-synonymous variants with a MAF < 3% in the East Asian panel of gnomAD. These gene pairs were also tested by four other methods using the same data. The *P* values of GxGrare were estimated based on $10^{6}$ permutations. The *P* values of PlinkEpi (min), FastEpistasis (min), and BOOST (min) represent the minimum calculated *P* values for all possible pairs of rare variants between the two genes using PlinkEpi, FastEpistasis and BOOST, respectively. /, no available *P* value was obtained in the analysis; Chr, chromosome number; AD, Alzheimer’s disease (with 246 cases and 172 controls in this dataset).

We also utilized PlinkEpi, FastEpistasis, BOOST, and GxGrare to analyze the top 10 interactions identified by iRUNNER using the same dataset (Table 3). FRG, FastKAST, and QuadKAST were excluded from this comparison due to abnormal crashes or difficulties in pre-defining the required sets under this analysis scenario. Among the ten gene pairs, GxGrare detected only one significant interaction, *HSP90AB1*–*WDR90* ($P_{\mathrm{GxGrare}}=2.7\times 10^{-3}$), that surpassed the Bonferroni-corrected threshold of $0.05/10=5\times 10^{-3}$. Eight out of 10 gene pairs failed to reach the significant level of 0.05 in GxGrare tests. In three SNP–SNP epistasis tests conducted with PLINK, BOOST achieved the lowest *P* value of 0.0129 for the rare variant pair rs200445657–rs376922153 between *TUBA3E* and *TUBB4B* genes. In contrast, the minimum *P* values obtained by PlinkEpi and FastEpistasis are 0.9876 and 0.1023, respectively. The results of PLINK further emphasize the limitations of SNP–SNP epistasis tests in detecting interactions involving rare variants. From another perspective, these findings also suggest that the significant interactions identified by iRUNNER may not be solely driven by individual variant pairs but could be the result of cumulative effects from multiple rare variant interactions between the two genes. Similar results were observed in other tested diseases (Table S7), highlighting the effectiveness of iRUNNER in identifying disease-associated rare variant interactions that might be missed by other methods.

#### Hirschsprung’s disease

HSCR is a rare and heterogeneous developmental disorder of the enteric nervous system (ENS), characterized by the absence of ganglia in the distal colon [62]. In this study, we analyzed a dataset comprising 443 Asian HSCR patients and 493 controls [50]. The most significant interaction in HSCR was between the *CDH23* and *MYO7A* genes, with a *P* value of $1.23\times 10^{-7}$ (BHFDR $q=5.35\times 10^{-3}$; Figure 3C; Table S7). *CDH23* is a member of the cadherin gene family, playing a crucial role in cell–cell adhesion. Abnormalities in neural crest cell development, including those seen in HSCR, often involve alterations in cadherin expression and function [63]. *MYO7A* encodes a myosin protein essential for anchoring cadherins to actin. Previous studies have reported a close interaction between *MYO7A* and *CDH23* in the human Usher syndrome [64], suggesting their potential interaction in HSCR. Among the top ten interactions identified by iRUNNER (Table S7), we observed three interactions: *EVT4–RET* ($P=1.60\times 10^{-5}$, q=0.232), *ROBO1–SEMA3D* ($P=3.48\times 10^{-5}$, q=0.289), and *ERBB4–RET* $(P=7.48\times 10^{-5}$, q=0.296), involving well-known HSCR risk genes *RET* and *SEMA3D* [62]. When examining the network formed by the 54 genes involved in the top 30 GGIs (Figure 3D), we found that these genes were tightly interconnected within a subnetwork (the left panel). Moreover, genes in the left subnetwork show highly relevant biological functions to HSCR, including processes such as “neural crest cell development (GO:0014032, $P_{\mathrm{GO}}=8.10\times 10^{-5}$, $q_{\mathrm{GO}}=4.80\times 10^{-3}$), differentiation (GO:0014033, $P_{\mathrm{GO}}=1.27\times 10^{-4}$, $q_{\mathrm{GO}}=5.88\times 10^{-3}$) and migration (GO:0001755, $P_{\mathrm{GO}}=1.75\times 10^{-5}$, $q_{\mathrm{GO}}=2.23\times 10^{-3}$)”, “regulation of nervous system development” (GO:0051960, $P_{\mathrm{GO}}=3.77\times 10^{-6}$, $q_{\mathrm{GO}}=8.33\times 10^{-4}$) and “neuromuscular process” (GO:0050905, $P_{\mathrm{GO}}=8.13\times 10^{-4}$, $q_{\mathrm{GO}}=0.014$). Interestingly, we observed that the genes within the right subnetwork primarily belong to the family of cytoskeletal proteins or basement membrane, which appear to be implicated in the colonization and differentiation of the ENS by regulating cell proliferation, cell-cell adhesion, cell migration, and cell projections [65,66].

All six genomic features in the iRUNNER regression model showed statistical significance (max $P=1.54\times 10^{-9}$; Table S8), collectively contributing to the approximately uniform distribution of *P* values observed in the HSCR dataset. The predictor of RVIB in controls demonstrated the weakest association (P=0.031). In the smaller AD dataset, this predictor failed to reach significance (P=0.399). While in the UC and CD datasets with larger (control) sample sizes, the predictor contributed significantly (UC: $P=4.30\times 10^{-4}$; CD: $P=2.57\times 10^{-5}$), albeit with modest coefficients ($\beta_{7}=0.082$ and $\beta_{7}=0.066$, respectively). These results indicate weak dependence of iRUNNER on control samples, particularly with limited controls. This finding also helps explain why the performance of iRUNNER is less affected when there is population stratification in controls, as mentioned earlier (Figure S4; Table S4).

#### Two major forms of inflammatory bowel diseases from the UKBB

**Figure 4:**
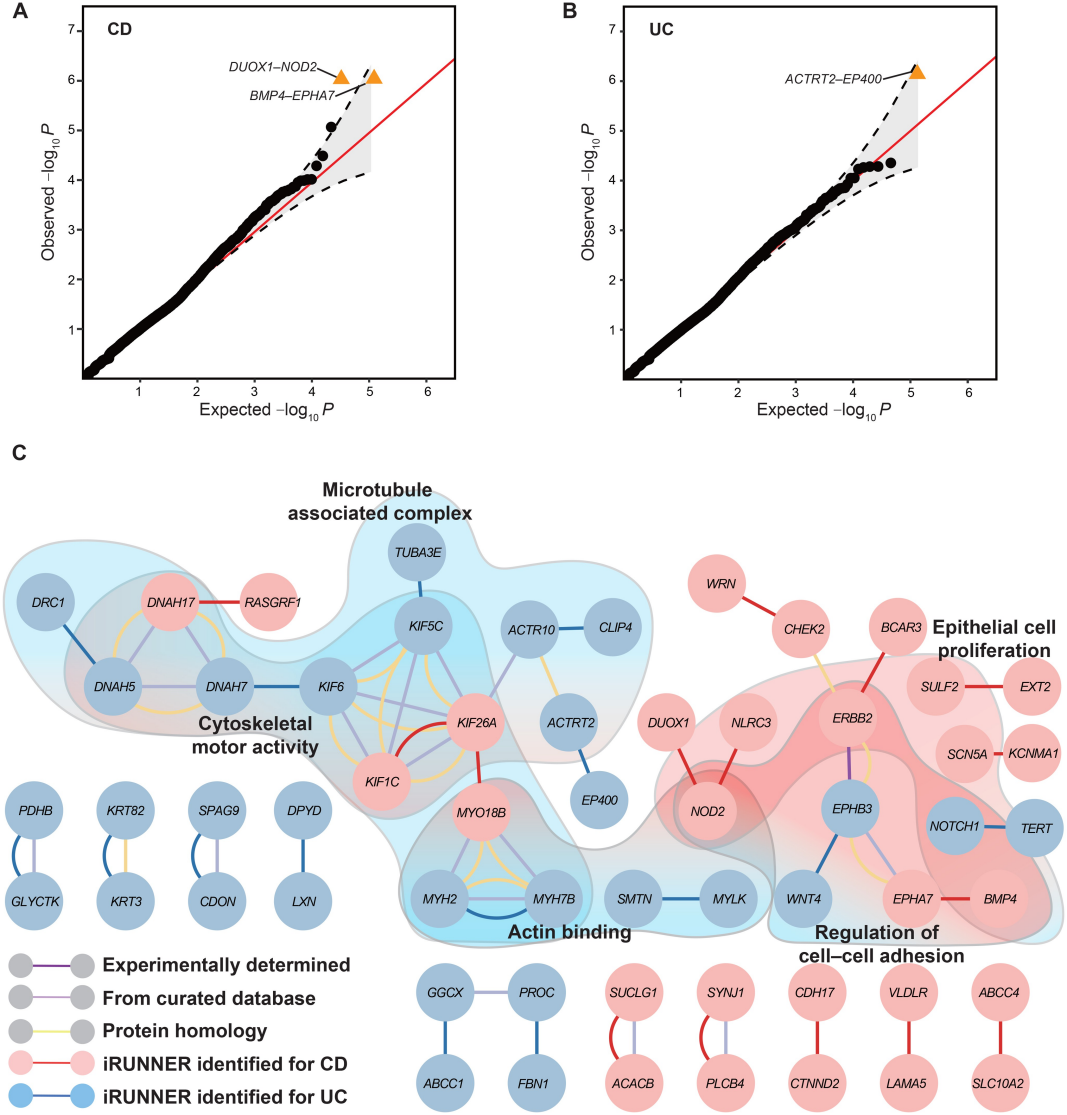
Discovery of GGIs associated with UC and CD by iRUNNER **A**. The QQ plot of *P* values generated by iRUNNER in CD (1605 cases, 1605 controls). **B**. The QQ plot of *P* values generated by iRUNNER in UC (3906 cases, 3906 controls). **C**. The network diagram of the top 15 interactions in the iRUNNER analysis for UC and CD. The blue and red nodes correspond to genes identified in UC and CD, respectively. The blue and red edges indicate interactions identified by iRUNNER in UC and CD, respectively. Other colored edges originate from interactions with confidence scores ≥ 0.5 in each STRING category (dark purple line for experimentally determined interactions; light purple line for interactions from curated databases; yellow line for protein homology), and text-mining connections are not considered. Nodes within the shaded areas indicate these genes are enriched in the corresponding GO functions (GO enrichment *q* < 0.05). UC, ulcerative colitis; CD, Crohn’s disease.

Ulcerative colitis (UC) and Crohn’s disease (CD) are two related yet different forms of inflammatory bowel diseases (IBDs). Here, we utilized UKBB WES data [51] to investigate GGIs associated with UC and CD using iRUNNER. By analyzing rare variants in 1605 cases with CD and 3906 cases with UC from England, along with an equal number of controls, iRUNNER identified three significant interactions associated with IBDs at a BHFDR q<5% (**Figure 4**A and B; Table S7). These included *DUOX1–NOD2* and *BMP4–EPHA7* for CD, as well as *ACTRT2–EP400* for UC. Specifically, for CD, iRUNNER identified *DUOX1–NOD2* as the most significant interaction ($P=8.71\times 10^{-7}$, q=0.024). *NOD2* is the first susceptibility gene identified for CD and plays a pivotal role in bacterial infection defense and intestinal epithelium barrier function [67]. Its interacting gene, *DUOX1*, has also been reported to be involved in innate immune responses to epithelial injury and microbial triggers [68]. Genes in another notable interaction, *BMP4* and *EPHA7* ($P=8.83\times 10^{-7}$, q=0.024), are implicated in maintaining intestinal barrier integrity and have been associated with chronic inflammatory diseases [69,70]. For UC, the most relevant interaction was *ACTRT2–EP400* ($P=7.03\times 10^{-7}$, q=0.048). *ACTRT2* encodes an actin-related protein that mediates cytoskeletal organization, a key process in maintaining intestinal epithelial barrier function [71]. The E1A binding protein 400 (*EP400*) is a variant chromatin-specific transcription factor that regulates gene activation [72]. Although there is direct or indirect evidence linking these genes to intestinal inflammation, to our knowledge, their interactions in the context of IBDs have not yet been revealed.

From the network perspective (Figure 4C), the top 15 gene pairs identified in UC were found to connect with those implicated in CD through well-documented interactions. Moreover, GO enrichment analysis indicated that genes prioritized for UC and CD could be enriched in the same GO functions. These findings are consistent with the understanding that susceptibility loci and pathways are shared between UC and CD [73]. However, we also noted that a large proportion of UC-associated genes enriched in the cytoskeletal-related functions of “cytoskeletal motor activity” (GO:0003774, $P_{\mathrm{GO}}=1.59\times 10^{-12}$, $q_{\mathrm{GO}}=3.01\times 10^{-10}$), “microtubule associated complex” (GO:0005875, $P_{\mathrm{GO}}=1.05\times 10^{-9}$, $q_{\mathrm{GO}}=1.41\times 10^{-7}$) and “actin binding” (GO:0003779, $P_{\mathrm{GO}}=3.68\times 10^{-4}$, $q_{\mathrm{GO}}=7.76\times 10^{-3}$). While the CD-associated genes are predominantly enriched in “epithelial cell proliferation” (GO:0050673, $P_{\mathrm{GO}}=3.11\times 10^{-4}$, $q_{\mathrm{GO}}=0.017$) and “regulation of cell-cell adhesion” (GO:0022407, $P_{\mathrm{GO}}=3.61\times 10^{-4}$, $q_{\mathrm{GO}}=0.017$). These distinct enrichment patterns suggest that the underlying pathogenesis pathways for UC may differ from those for CD.

#### Type 2 diabetes

Type 2 diabetes (T2D), also known as non-insulin-dependent diabetes mellitus, is a heterogeneous metabolic disorder characterized by impaired insulin secretion and insulin resistance [74]. In this analysis, a total of 34,847 T2D cases and 62,153 controls from the UKBB were included. iRUNNER was applied to examine 1,081,357 rare variants across 577,406 gene pairs and identified ten significant GGIs with BHFDR *q* < 0.05 (Figure S8). The results are summarized in Table S7. Many of these genes have been detected in previous studies from different omics perspectives. For example, a proteomic analysis [75] identified that junction plakoglobin (*JUP*) is strongly associated with diabetic retinopathy, a common secondary symptom in diabetes. Additionally, keratin 15 (*KRT15*) was implicated in diabetic keratopathy due to its role in corneal epithelium repair [76]. In our analysis, rare variant interactions between *JUP* and *KRT15* yielded a *P* value of $2.86\times 10^{-9}\;$($q=3.22\times 10^{-4}$). Another noteworthy interaction involved the gene pair *ACO1-DHTKD1*, which also achieved study-wide significance (*P* = 7.03 × 10^−8^, *q* = 4.74 × 10^−3^). Aconitase 1 (*ACO1*) plays a crucial role in cellular iron homeostasis and has been identified as a strong eQTL gene in pancreatectomized patients [77]. *DHTKD1* encodes a component of 2-oxoglutarate-dehydrogenase-complex-like protein and has been linked to T2D for regulating mitochondrial energy metabolism [78]. These findings highlight the effectiveness of iRUNNER in uncovering susceptibility rare variant interactions and pave the way to explore the molecular mechanisms of complex diseases and their complications.

### Computation cost

iRUNNER is implemented as a GGI analysis module within the KGGSeq software platform, a Java-based package designed for identifying genetic loci associated with human diseases or traits using high-throughput sequencing data. The computation time for iRUNNER depends on the sample size and the number of gene pairs being tested. All analyses in this study were performed on a server with an Intel Xeon Gold 6148 CPU (2.40 GHz) and 1 TB of memory. A complete iRUNNER analysis includes a series of steps: genotype loading, quality control, rare variant filtering, genomic feature annotation, and GGI testing. For testing interactions of 52,892 gene pairs in 246 cases and 172 controls, iRUNNER took approximately 18 min using 10 computing threads. Analyzing 117,012 gene pairs in 7812 individuals (equal number of cases and controls) required 2.28 h with the same computational resources (Table S3).

## Discussion

In this study, we introduce iRUNNER, a novel baseline mutation burden regression framework for analyzing GGIs between rare variants in exome-wide association studies of complex diseases. iRUNNER diverges from conventional case-control methods by assessing the enrichment of rare variant interactions between gene pairs within patient cohorts. This is achieved by evaluating the deviation of the observed burden of rare variant interactions in cases from the estimated baseline burden in the general population. Extensive simulations have demonstrated that iRUNNER outperforms state-of-the-art methods in statistical power and maintains robustness across sample sizes, epistatic models, and population structure of the controls. When applied to five representative complex diseases, iRUNNER has revealed a multitude of significant GGIs contributing to disease predisposition that would have been overlooked by conventional interaction tests. Notably, the identified gene pairs form intricate and highly interconnected networks, offering unparalleled insights into comprehending the mechanisms underpinning polygenic complex diseases.

The better performance of iRUNNER on rare variant interaction analysis is first attributed to its adoption of an appropriate estimation model. iRUNNER uses a truncated negative-binomial distribution-based regression model to predict the expected baseline burden of rare variant interactions for pairwise genes in the general population. A data-driven algorithm allows the model to choose an optimal truncation point, which is crucial for preventing potential model fitting jeopardization due to the fact that there are too many gene pairs with low interaction burdens in the real data. In the present study, iRUNNER has fitted the null model by regressing the RVIB of background gene pairs on six predictors derived from publicly available gene or variant annotations and one predictor from local control samples. We demonstrated that these predictors resulted in an approximately uniform distribution of *P* values in both simulated and real-data analyses. The significance of each predictor changes dynamically from sample to sample (Table S8). As a flexible model framework, iRUNNER also allows users to use additional predictors and make different predictor combinations.

The enhanced power of iRUNNER is also attributed to its functional weighting of rare variants. iRUNNER incorporates integrative functional prediction scores of rare variants to make a more informative RVIB in the regression model. The original functional score is a decimal number ranging from 0 to 1, which needs to be converted into an integer weight to apply to the truncated negative binomial regression model. To determine the optimal weighting scale, we conducted a grid search procedure to adjust the scale by minimizing the inflation index of MLFC. This approach contrasts with GxGrare, which arbitrarily uses 0 or 1 as weight values [22]. Moreover, our method does not presuppose a distribution for the functional scores, thereby circumventing the risk of extreme conservatism or optimism in testing results that can arise from incorrect assumptions about the probability distribution of these scores.

Besides serving as regression predictors and variant functional weights, knowledge from external databases such as STRING [79], BioGRID [80], and humanNet [81] can assist in selecting gene pairs that are more likely to be biologically relevant. This enables more efficient and effective analysis of GGIs in complex diseases [15]. In our study, iRUNNER used prediction scores from DIEP [41] by default to narrow all gene pairs down to a subset with potential digenic interaction effects on disease phenotypes. Users have the flexibility to employ other external databases for gene pair selection, as long as the data format adheres to a gene–gene-value three-column list. Such a filtering approach can significantly reduce multiple testing burdens and computational time in the exome-wide interaction analysis. The efficient computation of iRUNNER also benefits from the fact that it is a regression-based method. As a result, it can directly obtain *P* values without the need for permutation. For a dataset sequencing on 7812 individuals, iRUNNER finished rare variant interaction analysis for 117,012 gene pairs in 2.28 h with 10 threads (Table S3).

A hidden but important advantage of iRUNNER lies in its less sensitivity to the population structure of the controls. This attribute stems from the unique testing strategy employed by iRUNNER, which essentially calculates the relative difference between the observed RVIB and the estimated baseline RVIB at gene pairs in case samples. Consequently, the influence of population stratification in control samples on deviation calculations is small (Figure S4). The accurate estimation of baseline RVIB by iRUNNER is facilitated by utilizing frequency information from a reference cohort that is well-matched to the cases. When modeling gene frequency scores with data from ancestry-mixed controls (Figure S9; Table S9) or population-unmatched reference cohorts (Figure S10; Table S10), iRUNNER may exhibit an increased risk of type I error due to the selection of more significant gene pairs harboring population-private variants. Additionally, when causal variants were embedded into samples with ancestry-mixed controls (simulation details in File S1), iRUNNER yielded an average power of 78.5% (ranging 76%–81%; Table S11), demonstrating its high sensitivity for risk-enhancing interactions even under stratified controls. However, the complexity of population structure within case samples will weaken the ability of iRUNNER to detect substantial interactions, a challenge shared by other interaction analysis methods. Theoretically, the unique testing strategy enables iRUNNER to perform case-only analysis. However, in the absence of control subjects, iRUNNER tends to show an inflation of type I error with increasing sample size and a more lenient MAF threshold (Figure S3; Table S12), as the hard-filtering before interaction tests is not feasible to use control information. Therefore, case-only analyses with iRUNNER should be conducted with caution.

Our current work also has several limitations. First, the current framework is designed to analyze binary traits and cannot directly accommodate continuous traits. One possible workaround is categorizing continuous traits into cases and controls using a specific threshold. Second, this study focused primarily on non-synonymous rare variants. However, it is important to note that the iRUNNER framework is independent of mutation types. As public variant annotation databases continue improving, iRUNNER could potentially be extended for rare variants in non-coding regions. Given that genomic features of non-coding regions (*e.g.*, upstream, downstream, and intronic regions) may differ from those in coding regions, new predictors tailored to these non-coding variants would be needed. Third, the iRUNNER for interaction analysis is currently restricted to two-way tests between gene pairs. Detecting high-order interactions remains a significant challenge but represents a key area for future research. Fourth, iRUNNER is optimized to detect risk-enhancing effects and is insensitive to purely protective interactions, which manifest as depletion rather than enrichment in patients.

Despite these limitations, the proposed iRUNNER method offers a powerful and efficient tool for analyzing GGIs between rare variants. Its unique statistical strategy, which compares the observed interaction burden with the baseline burden, provides an additional approach for testing disease-associated interactions in binary traits. This attribute renders it particularly appealing for studies with limited sample sizes, where conventional approaches may lack statistical power. Remarkably, the gene networks formed by promising GGIs identified by iRUNNER hold substantial potential for uncovering the intricate molecular mechanisms underlying complex diseases.

## Ethical statement

Ethical approval for this study was obtained from the Ethics Committee of Zhongshan School of Medicine, Sun Yat-sen University, China (Approval No. 2022-009).

## Code availability

iRUNNER is implemented as a module of KGGSeq, available at http://pmglab.top/kggseq/. Source code and additional information to perform simulation and real case analyses are available at GitHub (https://github.com/pmglab/KGGSeq/). The code has also been submitted to BioCode at the National Genomics Data Center (NGDC), National Center for Bioinformation (CNCB) (BioCode: BT007786), which is publicly accessible at https://ngdc.cncb.ac.cn/biocode/tools/BT007786.

## CRediT author statement

**Hui Jiang:** Conceptualization, Methodology, Data curation, Formal analysis, Software, Funding acquisition, Validation, Visualization, Writing – original draft, Writing – review & editing. **Bin Tang:** Conceptualization, Methodology. **Kun Li:** Formal analysis, Validation. **Liubin Zhang:** Data curation, Methodology, Software. **Junhao Liang:** Methodology, Software. **Clara Sze-Man Tang:** Data curation, Resources. **Paul Kwong-Hang Tam:** Data curation, Resources. **Binbin Wang:** Data curation, Resources. **Youqiang Song:** Data curation, Resources. **Qiang Wang:** Data curation, Resources. **Mulin Jun Li:** Data curation, Resources. **Hailiang Huang:** Conceptualization, Writing – review & editing. **Miaoxin Li:** Supervision, Conceptualization, Methodology, Software, Funding acquisition, Resources, Validation, Writing – review & editing. All authors have read and approved the final manuscript.

## Competing interests

The authors have declared no competing interests.

## Supplementary Material

qzaf135_Supplementary_Data
