# Supplementary material for: iRUNNER: A Baseline Mutation Burden Regression for Identifying Gene Interaction Between Rare Variants for Diseases
Source: Genomics Proteomics Bioinformatics. 2025 Dec 30;23(6):qzaf135. doi: 10.1093/gpbjnl/qzaf135 (PMC13220759; doi:10.1093/gpbjnl/qzaf135)
Supplement: qzaf135_Supplementary_Data [file qzaf135_supplementary_data.zip › Supplementary material captions.docx]

**Supplementary material**

**File S1 Supplementary method**

**Figure S1 The density scatter plots of RVIB and the product of CDS lengths for pairwise genes**

**A.** Basic RVIB of all available gene pairs (301,457 pairs). **B.** Basic RVIB of gene pairs with a product of CDS length less than 500 (301,419 pairs). **C.** Functional weighted RVIB of all available gene pairs. **D.** Functional weighted RVIB of gene pairs with a product of CDS length less than 500. The RVIB scores were calculated based on 2000 pseudo-cases randomly drawn from the SG10K unrelated subjects. Gene pairs with a DIEP score over 0.8 and an RVIB score over zero were shown in the plots. Each point in the plot is colored by the number of neighboring points. RVIB, rare variant interaction burden; CDS, the coding region of a gene.

**Figure S2 The histogram of protein-coding gene pairs with a DIEP score over 0.5**

**Figure S3** **The QQ plots of *P* values generated by iRUNNER in balanced case-control and case-only samples**

**A.** iRUNNER was applied to samples with a varied number of cases, 500, 1000, 1500, and 2000, along with an equal number of controls or without controls, respectively. The MAF threshold in these analyses was set as 1%. **B.** iRUNNER was tested using different MAF cutoffs of 0.1%, 0.5%, and 5% in samples containing 2000 cases, along with an equal number of controls or without controls, respectively. The semi-simulation procedure produced the samples based on whole-genome sequencing data from unrelated SG10K subjects. The QQ plots were generated based on the results randomly selected once from 100 simulations in each group. MLFC, the mean log fold change.

**Figure S4 The QQ plots of *P* values generated by iRUNNER in** **ancestral mixed samples**

AFR-AMR in the table denotes that the pseudo-cases in this sample were from the AFR panel, while half of the controls were from the AFR panel, and the other half were from the AMR panel. Same as the other 19 group labels. In these analyses, the MAFs from the reference panel of gnomAD matched to the racial background of the cases (*e.g.*, African/African American panel of gnomAD for AFR-AMR) are used for rare variant selection and the accumulated MAF calculation. AFR, African; AMR, American; EAS, East Asian; EUR, European; SAS, South Asian panel from the 1000 Genome Project.

**Figure S5** **Power curves of iRUNNER and competing methods tested under varied MAF and OR of causal variants (**$\boldsymbol{=0.05}$**)**

The panels from left to right represent scenarios where the genotype OR of causal variants is 5 (**A**, **E**, **I**), 10 (**B**, **F**, **J**), 15 (**C**, **G**, **K**), and 20 (**D**, **H**, **L**), with a fixed sample size of 2000 cases and 2000 controls. In each plot, the power of the four methods was tested for assumed causal variants with MAF at 0.1%, 0.25%, 0.5%, 1%, and 2%. Samples were generated through a semi-simulation procedure based on whole-genome sequencing data from unrelated SG10K subjects. Power was estimated as the proportion of *P* values < 0.05 among 100 replicates.

**Figure S6 Power curves of iRUNNER and competing methods tested under varied OR and sample size (**$\boldsymbol{=}\boldsymbol{1\times}\boldsymbol{10}^{\boldsymbol{-6}}$**)**

Panels from left to right represent scenarios where the genotype OR of causal variants is 5 (**A**, **E**, **I**), 10 (**B**, **F**, **J**), 15 (**C**, **G**, **K**), and 20 (**D**, **H**, **L**), with a MAF of 1% and a fixed disease prevalence of 0.01. In each plot, the power of eight methods was tested at sample sizes of 1000, 2000, 3000, and 4000 under a balanced case-control design. Power was estimated as the proportion of *P* values $<1\times{10}^{-6}$ among 100 replicates.

**Figure S7 Power curves of iRUNNER and competing methods tested under varied MAF and OR of causal variants (**$\boldsymbol{=}\boldsymbol{1\times}\boldsymbol{10}^{\boldsymbol{-6}}$**)**

The panels from left to right represent scenarios where the genotype OR of causal variants is 5 (**A**, **E**, **I**), 10 (**B**, **F**, **J**), 15 (**C**, **G**, **K**), and 20 (**D**, **H**, **L**), with a fixed sample size of 2000 cases and 2000 controls. In each plot, the power of the four methods was tested for assumed causal variants with MAF at 0.1%, 0.25%, 0.5%, 1% and 2%. Power was estimated as the proportion of *P* values $<1\times{10}^{-6}$ among 100 replicates.

**Figure S8 The QQ plot of *P* values generated by iRUNNER in the type 2 diabetes dataset**

The QQ plot of *P* values generated by iRUNNER in type 2 diabetes (34,847 cases, 62,153 controls). The top two significant gene interactions are not shown in the plot; see details in Table S7.

**Figure S9 The QQ plots of *P* values generated by iRUNNER using MAF from ancestral mixed controls**

In these analyses, the MAFs from the reference panel of gnomAD matched to the racial background of the cases (*e.g.*, African/African American panel of gnomAD for AFR-AMR) are used for rare variant selection. The MAFs from the ancestral mixed controls are used for the accumulated MAF calculation.

**Figure S10 The QQ plots of *P* values generated by iRUNNER using MAF from ancestry-unmatched reference**

In these analyses, the MAFs from the reference panel of gnomAD matched to the racial background of the half ancestral mixed controls (*e.g.*, the Latino (AMR) panel of gnomAD for AFR-AMR) are used for rare variant selection and the accumulated MAF calculation.

**Table S1 Information on 19 genes pre-excluded before type Ⅰ error simulations**

**Table S2 Information on assumed risk rare variants in power simulation experiments**

**Table S3 Real high-throughput sequencing datasets used in the present paper**

**Table S4 Empirical type Ⅰ error rates of iRUNNER in samples with ancestral mixed controls**

**Table S5 Statistical power of iRUNNER and competing methods**

**Table S6 The average power ± SD of iRUNNER and competing methods**

**Table S7 Gene-gene interaction analysis on rare variants using iRUNNER and competing methods**

**Table S8 The estimated parameters of seven predictors by iRUNNER**

**Table S9 Empirical type Ⅰ error rates of iRUNNER using MAF from ancestral mixed controls**

**Table S10 Empirical type Ⅰ error rates of iRUNNER using MAF from ancestry-unmatched reference**

**Table S11 Statistical power of iRUNNER in samples with ancestral mixed controls**

**Table S12 Empirical type Ⅰ error rates of iRUNNER in case-only samples**
