# Supplementary figures and images for: iRUNNER: A Baseline Mutation Burden Regression for Identifying Gene Interaction Between Rare Variants for Diseases
Source: Genomics Proteomics Bioinformatics. 2025 Dec 30;23(6):qzaf135. doi: 10.1093/gpbjnl/qzaf135 (PMC13220759; doi:10.1093/gpbjnl/qzaf135)

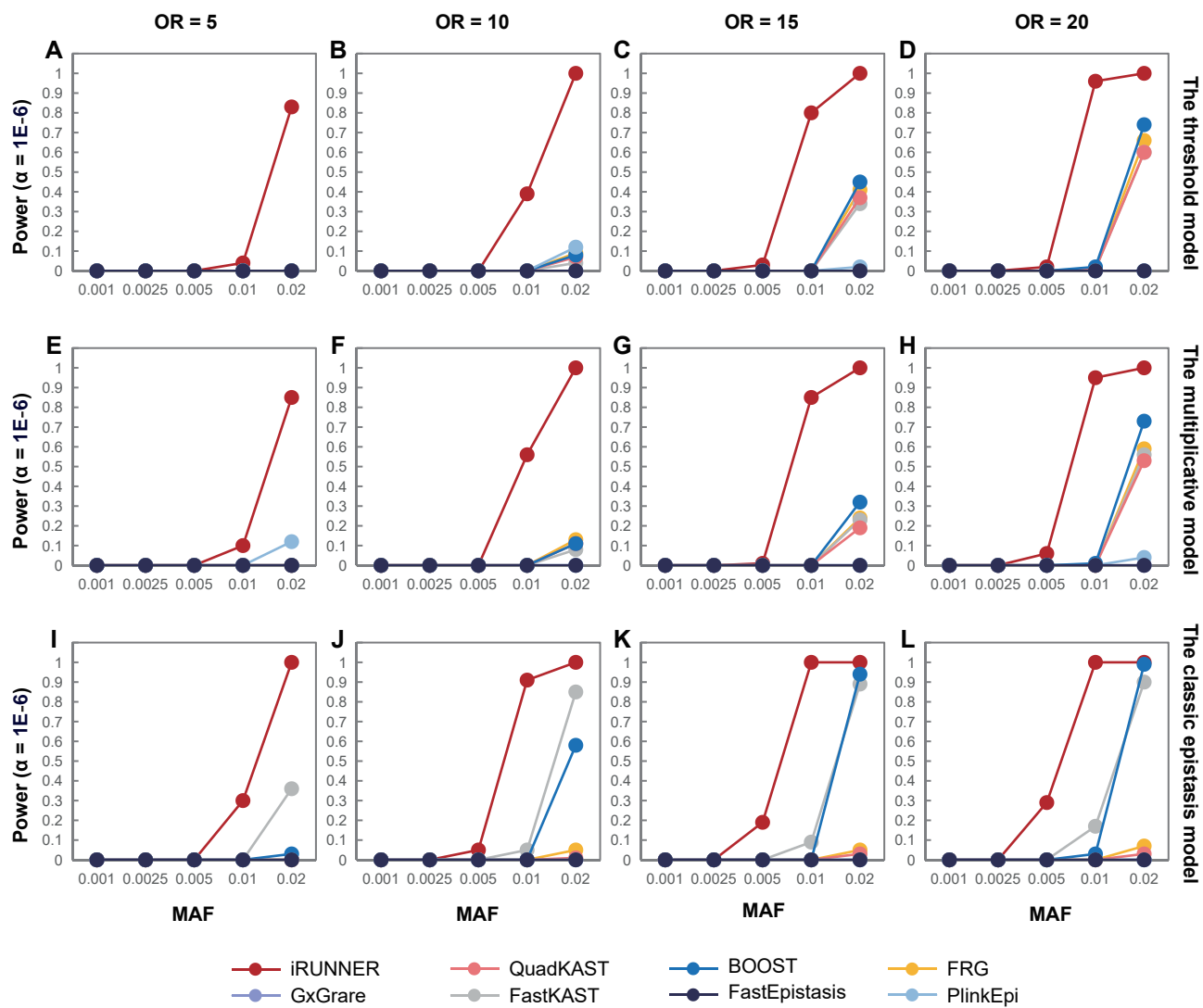

Supplement: qzaf135_Supplementary_Data [file qzaf135_supplementary_data.zip › FigureS7.pdf]

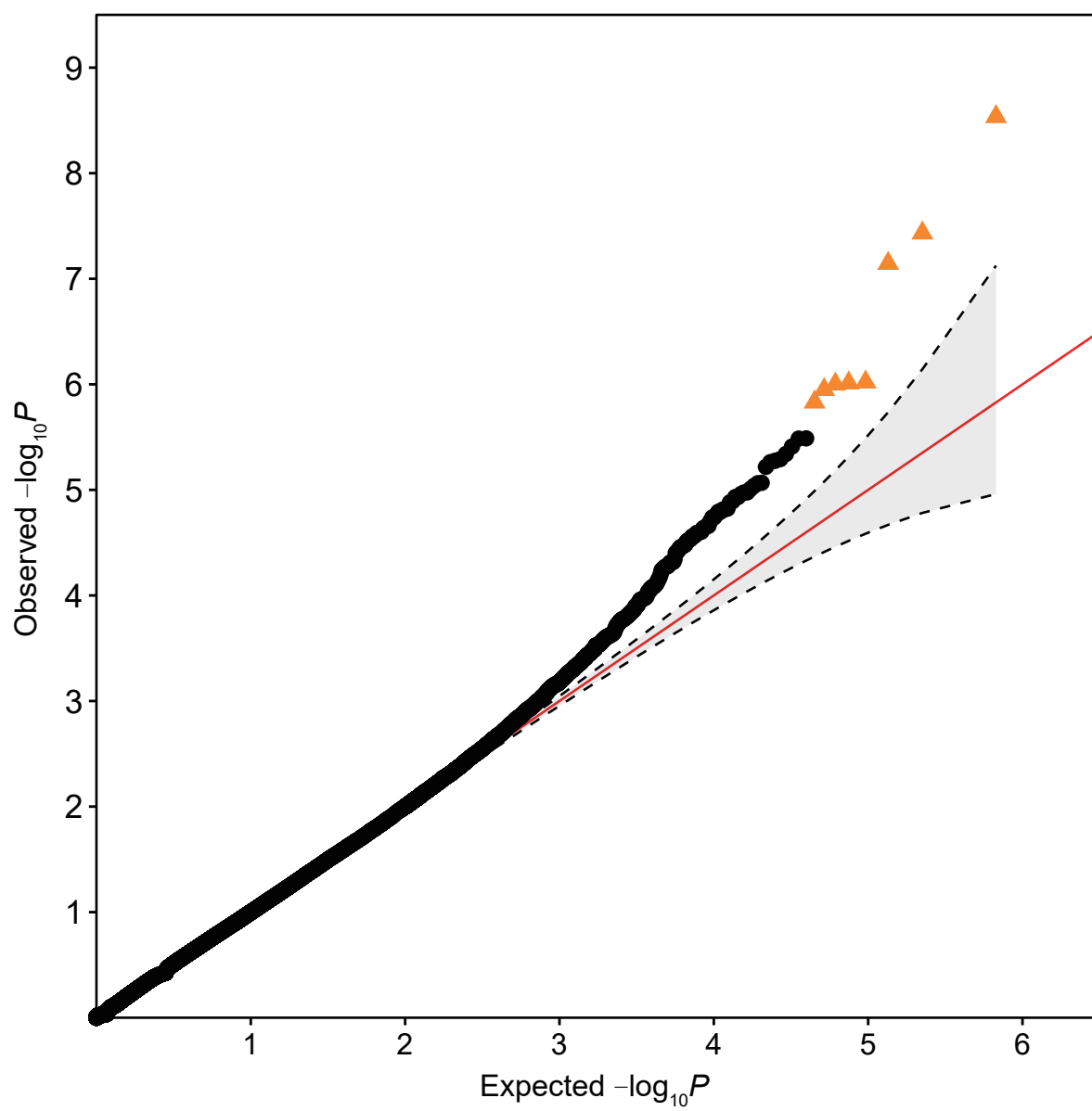

Supplement: qzaf135_Supplementary_Data [file qzaf135_supplementary_data.zip › FigureS8.pdf]

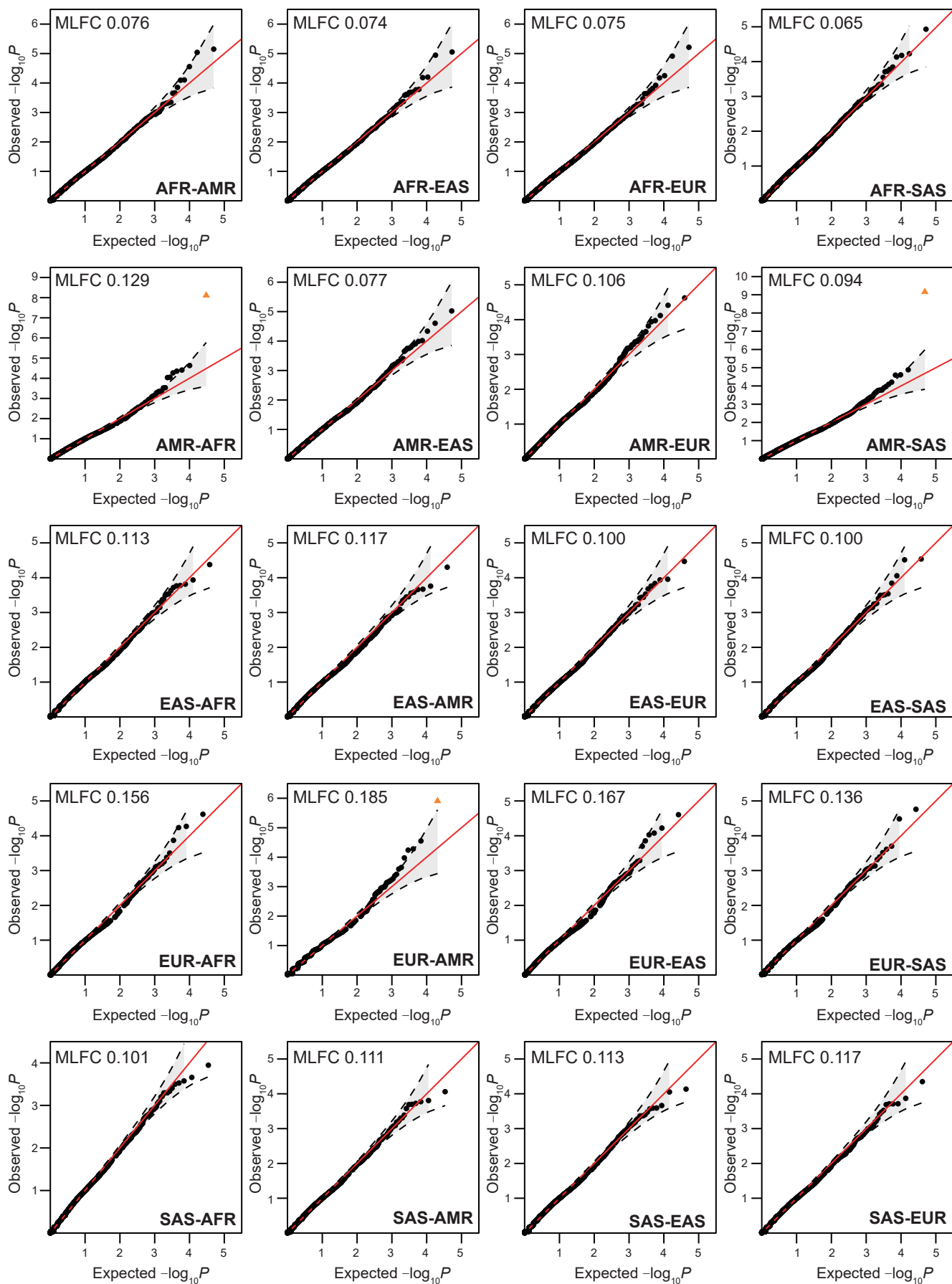

Supplement: qzaf135_Supplementary_Data [file qzaf135_supplementary_data.zip › FigureS9.pdf]

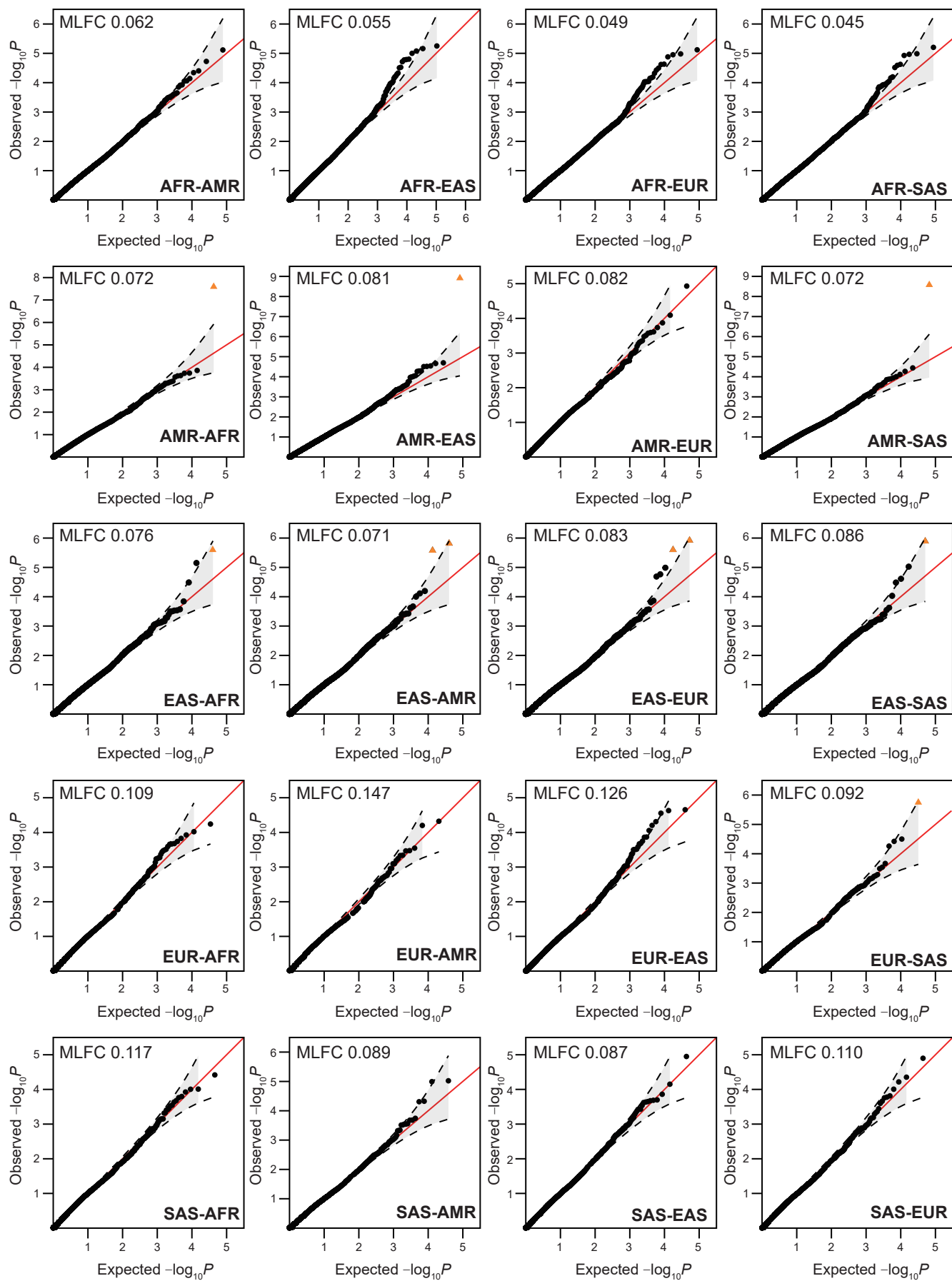

Supplement: qzaf135_Supplementary_Data [file qzaf135_supplementary_data.zip › FigureS10.pdf]

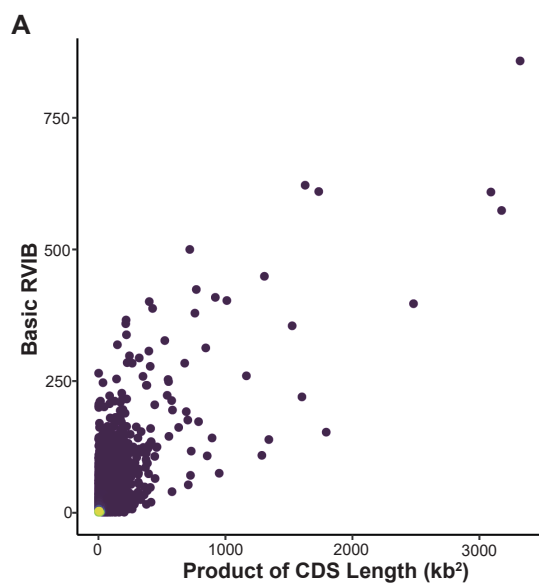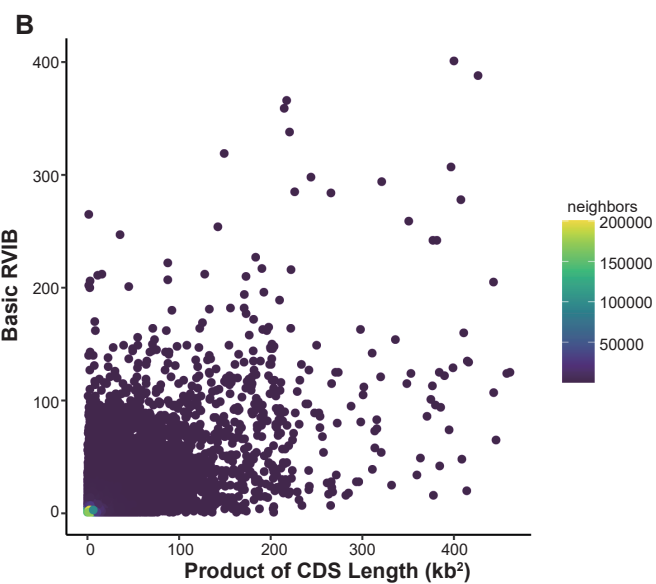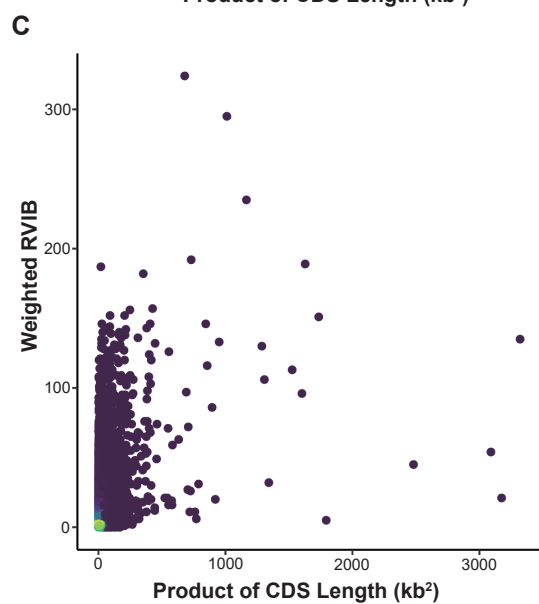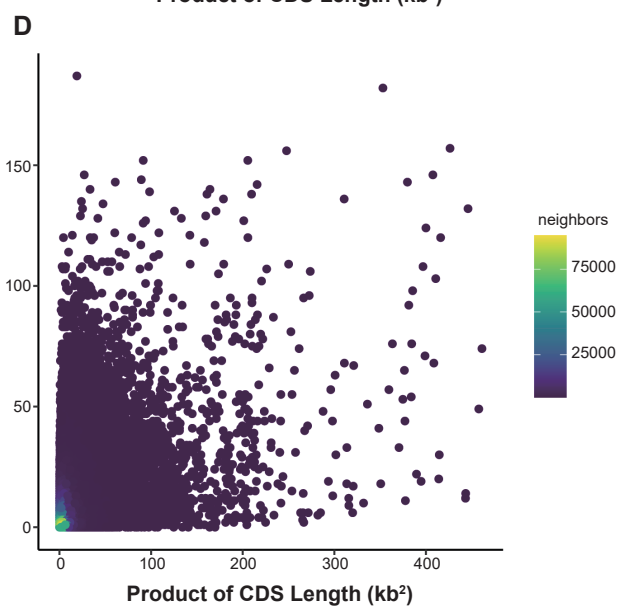

Supplement: qzaf135_Supplementary_Data [file qzaf135_supplementary_data.zip › FigureS1.pdf]

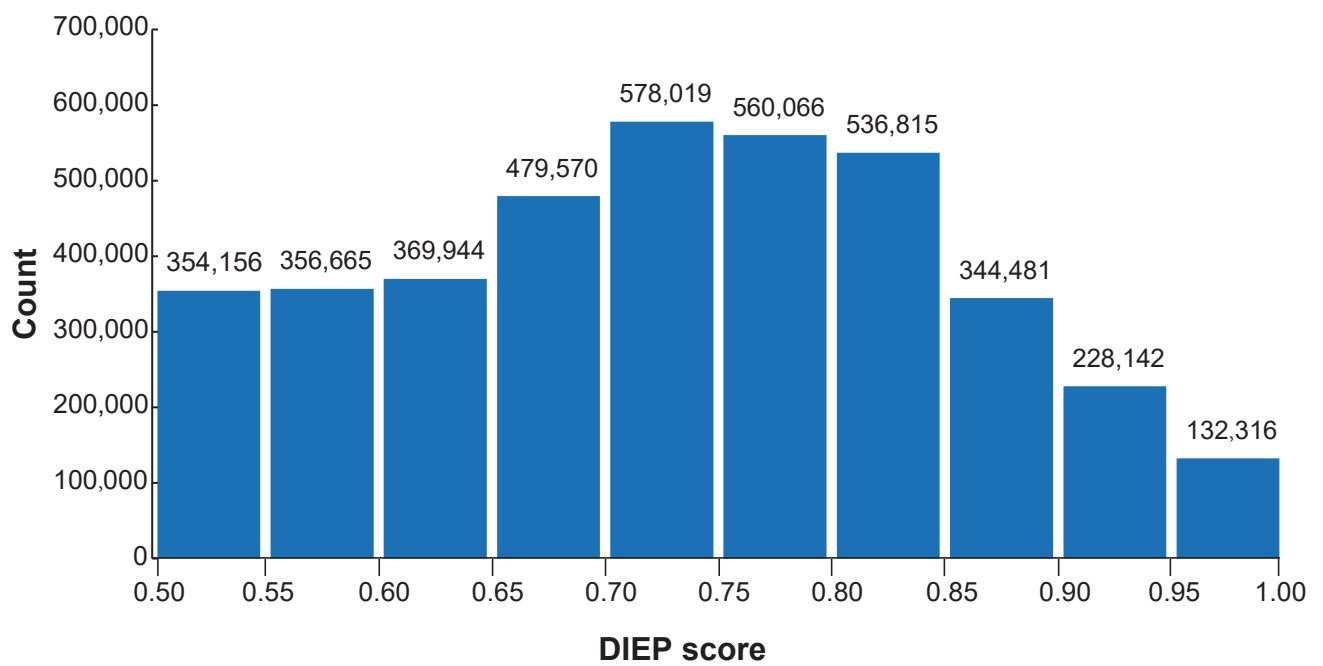

Supplement: qzaf135_Supplementary_Data [file qzaf135_supplementary_data.zip › FigureS2.pdf]

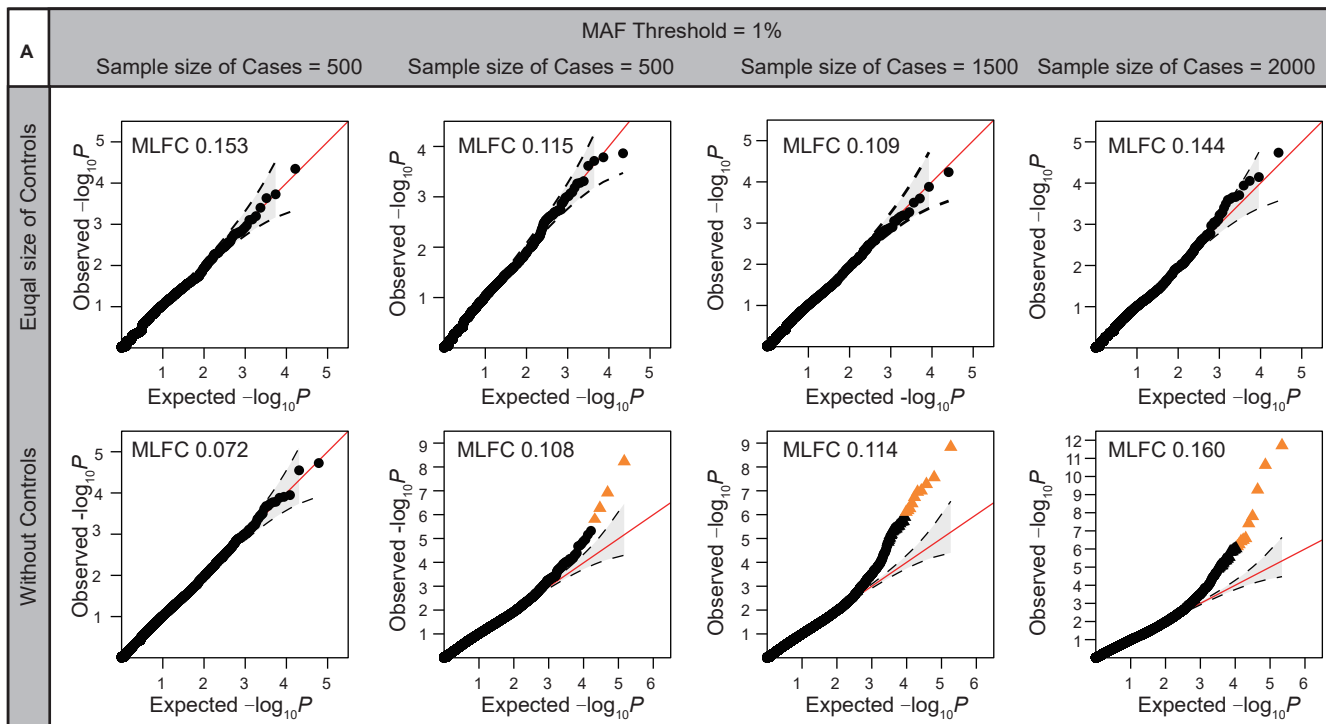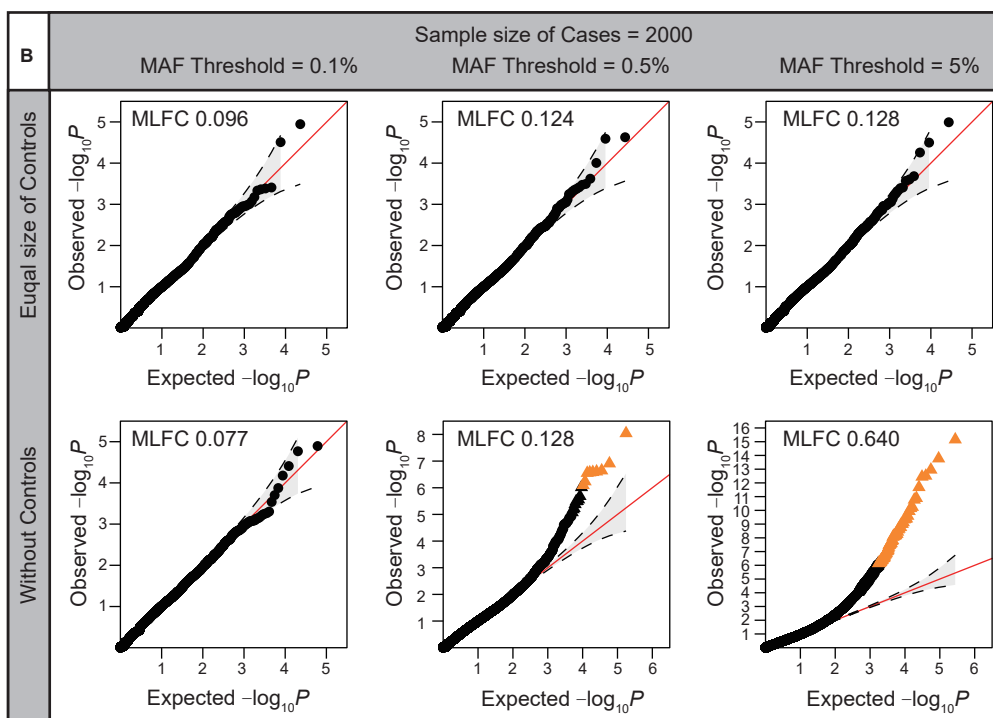

Supplement: qzaf135_Supplementary_Data [file qzaf135_supplementary_data.zip › FigureS3.pdf]

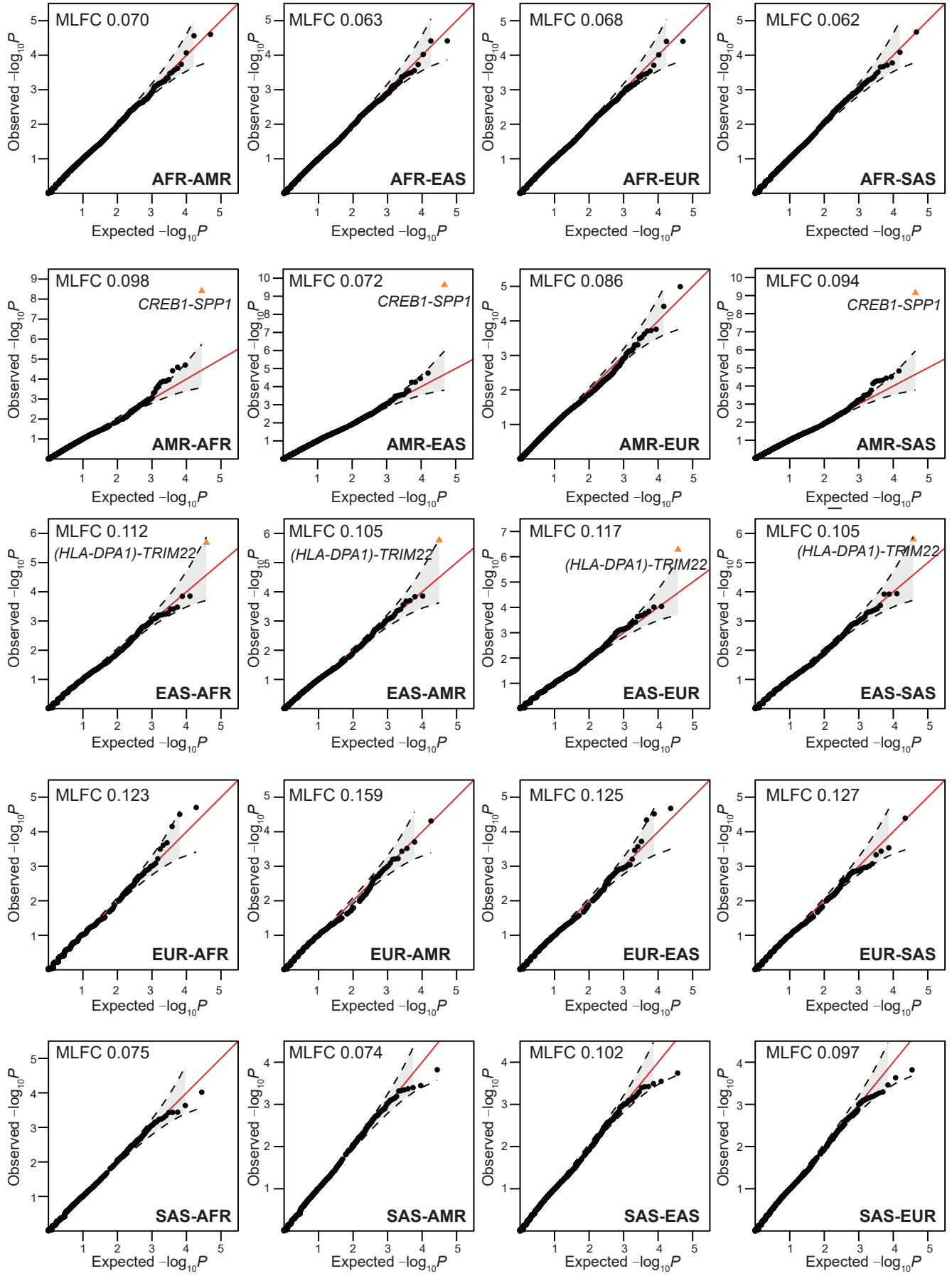

Supplement: qzaf135_Supplementary_Data [file qzaf135_supplementary_data.zip › FigureS4.pdf]

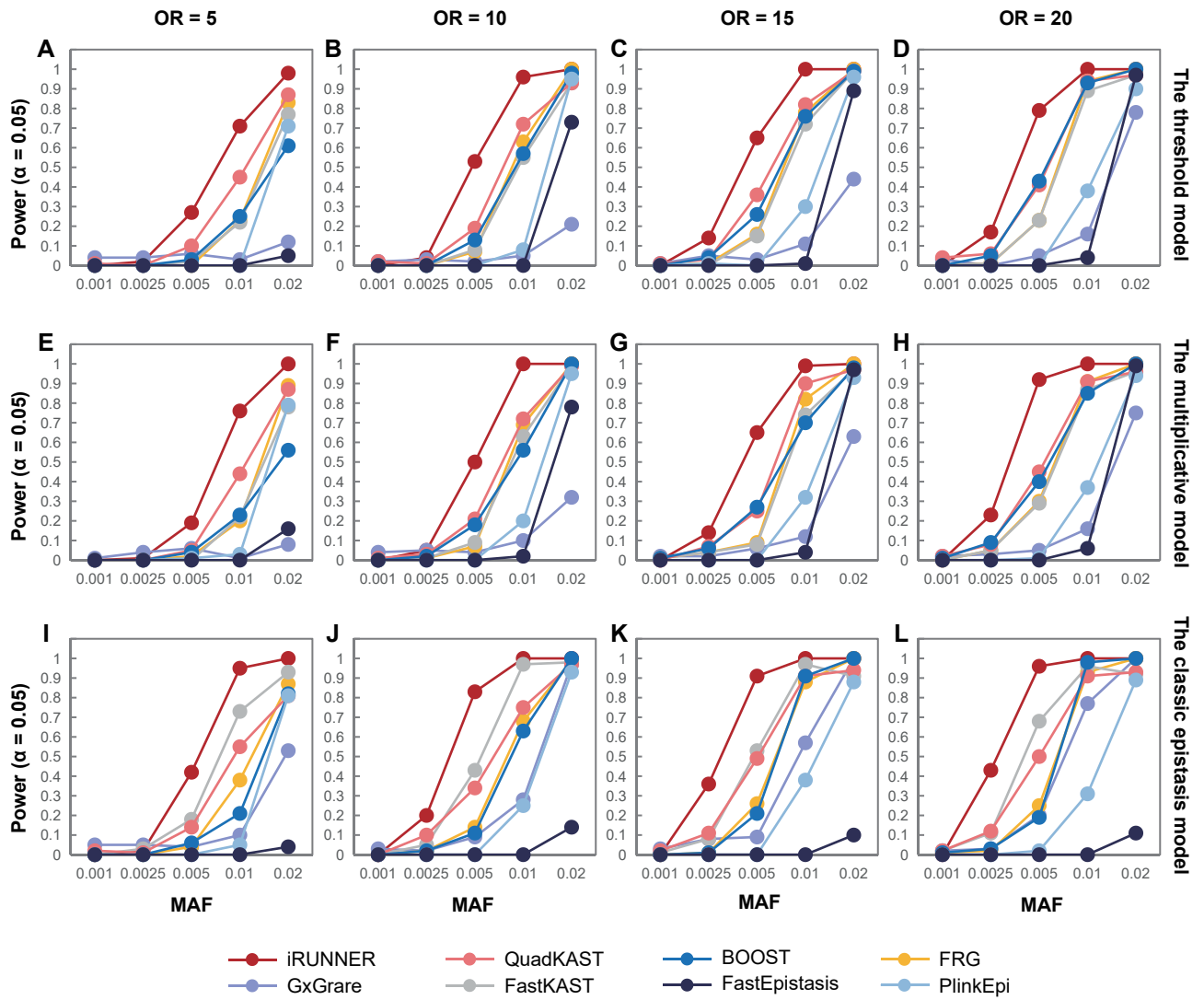

Supplement: qzaf135_Supplementary_Data [file qzaf135_supplementary_data.zip › FigureS5.pdf]

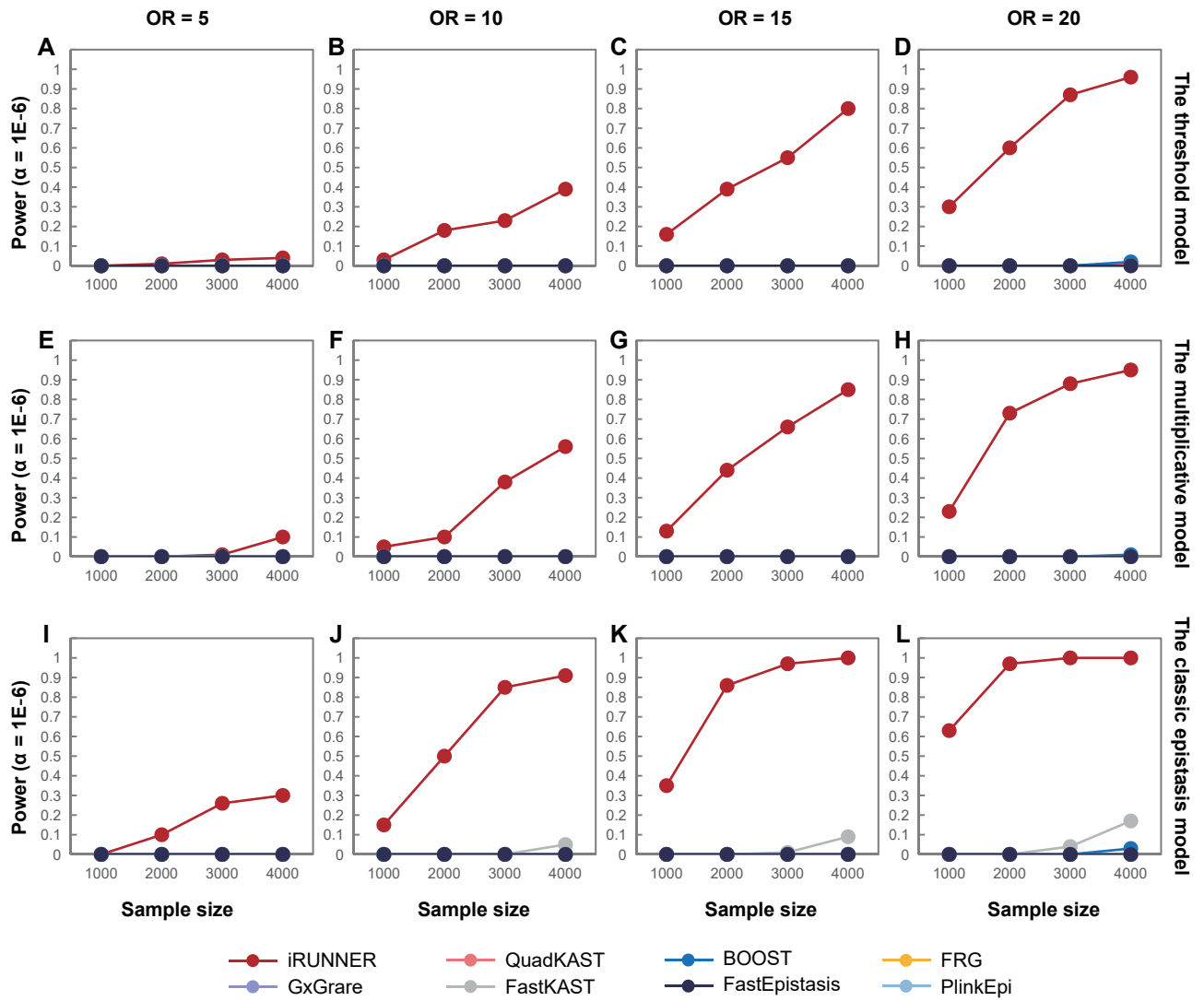

Supplement: qzaf135_Supplementary_Data [file qzaf135_supplementary_data.zip › FigureS6.pdf]
